# The effects of oral cannabidiol supplementation on blood pressure in adults: a systematic review of randomised controlled trials

**DOI:** 10.1186/s42238-026-00463-3

**Published:** 2026-06-19

**Authors:** Albert Roberts, Solon Fountzopoulos, Terun Desai

**Affiliations:** Institute of Sport, Exercise and Health, Division of Surgery and Interventional Science, University College London, ISEH, 170 Tottenham Court Road, London, W1T 7HA UK

**Keywords:** Cannabidiol, CBD, Blood pressure, Hypertension, Cardiovascular health, Dietary supplements

## Abstract

**Background:**

Hypertension is a leading risk factor for cardiovascular disease, particularly in ageing populations. While pharmacological interventions are common, issues with long-term adherence and side effects have prompted interest in alternative treatments. Cannabidiol (CBD), a non-psychoactive component of *Cannabis sativa*, has been proposed as a potential agent for blood pressure regulation due to its anxiolytic, anti-inflammatory, and vasodilatory properties. This systematic review aimed to evaluate the effects of oral CBD supplementation on blood pressure in adults with normotension and hypertension.

**Methods:**

A systematic search of PubMed, Web of Science, Scopus, and Medline was conducted in September 2024. Eligible studies were randomised controlled trials (RCTs) involving oral CBD administration in normotensive or hypertensive adults, with blood pressure as an outcome. Studies involving animals, inhaled CBD, or non-English texts were excluded. Risk of bias was assessed using the Cochrane Risk of Bias tool. Clinical heterogeneity was assessed by comparing study populations, CBD dosing regimens, outcome measures, and assessment conditions. Substantial variability in dosing and blood pressure outcome reporting precluded quantitative pooling; therefore, results were synthesised narratively due to clinical heterogeneity.

**Results:**

Four RCTs involving 120 participants met the inclusion criteria. Studies varied in CBD dose (225–600 mg/day), duration (two hours to 5 weeks), and participant health status. An association was observed between CBD dosage (mg/day) and reductions in blood pressure indicators, with greater reductions occurring at higher doses. All four studies reported statistically significant reductions in systolic blood pressure compared to placebo, particularly under stress or during sleep. Two studies reported lower diastolic pressure. The strongest effects were observed with acute administration of the highest-dose studies of 600 mg/day. Side effect severity was generally mild to moderate, including nausea, diarrhoea, and fatigue. No serious cardiovascular events were reported.

**Conclusion:**

Oral CBD may reduce blood pressure amongst healthy and hypertensive individuals, particularly under stressful conditions and during sleep. Limitations included small sample sizes, short trial durations, variability in CBD matrices and dosages, lack of pharmacokinetic data, and uncertainty surrounding hepatic safety. Larger, longer-term trials with homogeneous supplementation strategies and bioavailability measures are needed to determine CBD’s therapeutic role in blood pressure management.

**Supplementary Information:**

The online version contains supplementary material available at https://doi.org/10.1186/s42238-026-00463-3.

## Background

Hypertension is defined as a blood pressure of ≥ 140 mmHg systolic or ≥ 90 mmHg diastolic (World Health Organization 2023). Both elevated systolic and diastolic pressures contribute to adverse cardiovascular outcomes, with systolic pressure being a slightly greater predictor (Flint et al. 2019). Both systolic and diastolic pressures can be combined into mean arterial pressure, a composite indicator of organ perfusion (DeMers and Wachs 2023). The World Health Organization (World Health Organization 2023) estimates that achieving 50% global hypertension control by 2050 could prevent 76 million deaths and 450 million disability adjusted life-years (DALYs). However, pharmacological treatment alone is insufficient to achieve population-level blood pressure control. Achieving this target will require improved adherence to existing treatments, expansion and inclusion of holistic lifestyle interventions, and implementation of adjunctive approaches (Mancia et al. 2023). Furthermore, the economic benefits would outweigh costs by up to 18 to 1 despite substantial implementation expenses (World Health Organization 2023). Although hypertension is starting to decrease in high-income countries, it is predicted to rise in some middle-income, and mainly low-income countries contributed by increased dietary sodium intake with lack of early treatment (Boateng and Ampofo 2023). Hypertension affects cardiac remodelling which can lead to hypertrophy and fibrosis of cardiac tissue, increasing the risk for heart failure, with hypertensive heart diseases increasing globally with the ageing population (Gallo and Savoia 2024). Additionally, resistant hypertension is rising, leading to chronic elevation of blood pressure with major complications like stroke and myocardial infarction (Dzau and Hodgkinson 2024). Resistant hypertension may benefit most from pharmacological treatment in the short-term, but long-term effects may be uncertain due to poor compliance from patients (Tian et al. 2024). Thus, alternative strategies which can be embedded into the lifestyle are required.

Cannabidiol (CBD) is a major phytocannabinoid found in Cannabis sativa, with its abundance relative to delta-9-tetrahydrocannabinol (THC) dependent on the plant chemotype; CBD has gained significant attention for its potential cognitive benefits, including reductions in stress and anxiety (Moltke and Hindocha 2021). However, CBD has been shown to induce vasodilation in animal models (Atalay et al. 2019). CBD also has antioxidant properties that can modulate redox system and benefit inflammatory conditions including cardiovascular disease (Atalay et al. 2019). This paper evaluates the vascular effect of CBD on blood pressure in human participants.

Unlike THC, CBD is non-psychoactive and does not induce euphoria, making it a popular therapeutic option. Its biological mechanisms of action involve interactions with the endocannabinoid system, modulation of serotonin receptors, and anti-inflammatory properties (De Almeida and Devi 2020), suggesting that it could help regulate blood pressure. In the UK, it is recommended to limit daily consumption to 10 mg (Food Standards Agency and Food Standards Scotland Update Consumer Advice for CBD [press release], 2023). However, recommended daily intakes vary by country and therefore this review will explore doses between 225–600 mg a day, evaluating the effects of CBD on systolic and diastolic blood pressures (including mean arterial pressure) and self-reported physiological tolerance and safety to CBD consumption in humans.

Preclinical studies have provided some evidence supporting the anti-hypertensive effects of CBD. For instance, various in vitro and in vivo models have demonstrated that CBD can induce vasodilation, reduce cardiac contractility, and lower blood pressure in response to stress (Garza-Cervantes et al. 2020). Additionally, CBD's antioxidative and anti-inflammatory properties (Atalay et al. 2019) may contribute to its potential protective effects against the development of hypertension and related cardiovascular complications. However, the translation of these findings to human populations remains unclear, with clinical trials yielding mixed results.

Human studies on the effects of CBD supplementation on hypertension are limited but growing. The evidence suggests beneficial effects of CBD on reducing elevated blood pressure, with all studies included in the review reporting significant reduction of systolic blood pressure (Jadoon et al. 2017; Sultan et al. 2020; Dragun et al. 2023; Dujic et al. 2023). However, the various magnitude of blood pressure reduction can be attributed to variations in study design, sample size, dosage, and the health status of participants. While generally well-tolerated, CBD has been linked to elevated liver enzymes, sedation, and gastrointestinal effects, with risks increasing at higher or prolonged doses, though no clear safety threshold has been established in humans (Gingrich et al. 2023). Another concern is the ability of CBD to inhibit the cytochrome P450 enzyme system, which can lead to altered metabolism of various drugs, potentially resulting in adverse drug interactions (Millar et al. 2019).

Recent population-based evidence indicates substantial CBD exposure in Europe, with approximately 10% of adults reporting use in France (Casanova et al. 2022), while population-level research across the region remains limited (Fortin et al. 2025). Currently in the UK, CBD has been approved for only a limited number of conditions (Medicines and Healthcare products Regulatory Agency 2016), such as certain forms of epilepsy (National Institute for Health and Care Excellence, 2019). Consequently, CBD is widely used in a largely self-directed manner, often by health conscious individuals (Fortin et al. 2025). Importantly, CBD is often used for motivations overlapping those of THC, such as stress reduction and sleep improvement, but without psychoactive effects. Evidence indicates that CBD is frequently used as a substitute or complementary to THC, to mitigate THC-related harms, with co-users reporting shared behavioural motivations (Fortin et al. 2025).

Despite this widespread use, evidence supporting CBD’s cardiovascular effects remains limited, in part because clinical research on CBD and herbal cannabis has been structurally delayed by legal and regulatory restrictions (Piomelli et al. 2017), as well as economic and technical constraints (Fortin and Massin 2020). These barriers have contributed to a fragmented evidence base despite growing population-level exposure.

This systematic review aimed to critically evaluate the existing literature on the effects of oral CBD on blood pressure. By synthesising findings from randomised control trials, this review seeks to explore the potential role of CBD in blood pressure management. Specifically, the review will address the following research questions:

1. Does CBD supplementation effectively reduce blood pressure in individuals with normotension and hypertension?
2. Are there any adverse effects associated with the use of CBD for hypertension?

## Materials and methods

A systematic search was conducted across four databases: PubMed, Web of Science, Scopus, and Medline in September 2024. No restrictions were placed on publication year besides an English language requirement. Search terms were combined using Boolean operators and focused on titles and abstracts (refer to Supplement 1 for the specific search syntax and all reports sort for retrieval).

After conducting the search and removing duplicates, two authors (AR and SF) independently screened the titles and abstracts. Any differences in their assessments were resolved through discussion, with a third reviewer (TD) consulted if needed. From this process, 79 studies were selected for full-text review. These full-text articles were then evaluated for eligibility based on the pre-defined criteria. After full-text screening, there was unanimous agreement among the three authors (AR, SF, and TD), resulting in four articles being included in the review. The eligibility criteria are detailed in Table 1.

**Table 1:** Study eligibility criteria

	Inclusion	Exclusion
Trial design	Human studies Randomised controlled trials (parallel/crossover assignment with placebo control) Single/double/triple blinding	Animal studies Research letters
Participants	Healthy Individuals with normotension, or hypertension	Individuals with heart failure, coronary artery disease, arrhythmias, stroke/transient ischemia, peripheral arterial disease, congenital heart disease, valvular heart disease
Intervention	Acute/chronic oral administration of CBD	Administration of THC, inhaled administration of CBD, over the counter (OTC) drugs
Outcomes	Systolic blood pressure/Diastolic blood pressure/Mean arterial pressure	Endocannabinoid-related outcomes were defined as biochemical or molecular measures of endocannabinoid system activity (e.g. plasma anandamide levels or CB1/CB2 receptor expression)
Comparison	Placebo control	No comparison group
Publication type	Full articles in peer-review journals Written in English	Abstracts, case studies/series, non-peer-reviewed articles, study protocols, non-English language articles

### Data extraction

Key information was systematically extracted from the final set of eligible studies. This included outcome measures such as mean arterial pressure, systolic blood pressure, and diastolic blood pressure, along with their associated time points and analyses. For each outcome, all available data across relevant time points and analysis methods were sought. To ensure accuracy and consistency, the extracted data were reviewed and verified by all authors. The primary researchers (AR and SF) were responsible for gathering all qualitative data, including details such as the type of clinical trial, the investigational product or treatment, the duration and route of administration, and participant characteristics.

### Data preparation

No additional data transformations were required. All blood pressure outcomes and adverse events were extracted as reported in the included studies, with no imputation of missing data or conversion of summary statistics.

### Quality assessment and risk of bias

To ensure a thorough evaluation of the potential limitations in the included studies and to derive reliable conclusions, and in line with Cochrane Collaboration Guidelines (van Tulder et al 1976), two authors (AR and SF) independently assessed each study's methodological quality and risk of bias (Supplement 2). Any disagreements were resolved through a third-party evaluation by TD. The Cochrane Risk of Bias 2 tool encompasses several domains: 1) selection bias (including random sequence generation and allocation concealment), 2) performance bias (blinding of participants and personnel), 3) detection bias (blinding of outcome assessment), 4) attrition bias (incomplete outcome data), 5) reporting bias (selective reporting), and 6) other bias (additional sources of bias). Each study's risk of bias was categorized as low risk (where bias is unlikely to substantially affect the results), unclear risk (where bias may raise some concerns about the results), or high risk (where bias significantly undermines confidence in the results).

### Synthesis

Clinical heterogeneity was evaluated by grouping studies according to their specific indications and measured outcomes, including condition-specific endpoints and reported adverse events. While the method of CBD administration was consistent, variations in dosing and outcome reporting contributed to heterogeneity, limiting the feasibility of a meta-analysis. For studies employing weight-based CBD dosing (e.g., Dujic et al.), outcomes were synthesised at the study level using reported follow-up time points, without stratification by individual dose, as dose-specific outcome data were not reported. For all continuous outcomes the effect measure used was the mean difference (MD) with 95% confidence intervals (CI).

Study characteristics were tabulated in the master table (Table 2) and compared against the review objectives. To determine which studies contributed to each synthesis, trials were further stratified by population health status (healthy vs hypertensive), dosing regimen (acute vs chronic), and CBD formulation (standard vs DehydraTECH™). DehydraTECH™ is a lipid-based capsule designed to enhance bioavailability of CBD (Dujic et al. 2023). This approach ensured that only studies with comparable populations, interventions, and outcome measures were synthesised together, in line with the review objectives.

**Table 2:** Summary of clinical studies evaluating cannabidiol (CBD) effects on blood pressure (BP)

Study	Population	Sample (n)	Design	Trial Length & Regimen	CBD Dose (mg)	Primary Outcomes (timing & measure)	Incidence of adverse events n (%)
Jadoon et al. (2017)	Healthy volunteers	9 M *Initiated with 10 but 1 man withdrew for personal reasons before study started	Randomised, placebo-controlled, double-blind, crossover	2 h (Acute) – clinic & stress tests	600 mg single dose	Resting: SBP ↓ 6 mmHg [95% CI − 12 to − 1], p < 0.05; DBP ↓ 5 mmHg [95% CI − 9 to − 1], *p* < 0.05 Isometric handgrip stress: SBP ↓ 6 mmHg [95% CI − 10 to − 1], *p* = 0.001 Mental arithmetic stress: SBP ↓ 8 mmHg [95% CI − 12 to − 4], *p* < 0.01; DBP ↓ 6 mmHg [95% CI − 11 to − 2], *p* < 0.01 Cold pressor stress: SBP ↓ 8 mmHg [95% CI − 12 to − 4], *p* < 0.01; MAP ↓ 5 mmHg [95% CI − 9 to − 2], *p* < 0.05	None reported
Sultan et al. (2020) (Acute phase)	Healthy volunteers	26 M	Randomised, placebo-controlled, double-blind, parallel	Day 1 (Acute) – clinic BP up to ~ 3 h	600 mg single dose (day 1 of 7-day course)	Resting: MAP ↓ 2 mmHg [95% CI − 3.6 to − 0.3], *p* = 0.04 Stress: SBP ↓ 6 mmHg [95% CI − 10 to − 1], *p* = 0.001 (minute‑specific: − 6.6 mmHg [95% CI − 12.7 to − 0.4] at 2 min; − 6.5 mmHg [95% CI − 12.7 to − 0.3] at 3 min)	Adverse events were not reported separately for the acute assessment phase; see Sultan et al. (2020) chronic phase study row
Sultan et al. (2020) (Chronic phase)	Healthy volunteers	26 M	Same as above	Days 6–7 (Chronic) – 24-h ABPM (day 6) + clinic/stress (day 7)	600 mg/day × 7 days	24‑h ABPM (day 6): NS (95% CI NR) Stress (day 7): SBP ↓ 5.7 mmHg [95% CI − 10 to − 1], *p* = 0.02; resting clinic effect not maintained	Adverse events were reported across the 7-day study period: CBD group — lack of appetite day 4, headache day 3, insomnia days 2–3, hyperactivity days 2–3, dysuria days 5–6; placebo — migraine day 4, light-headedness day 6. Events were not formally attributed to acute vs chronic phase
Dragun et al. (2023)	Untreated hypertension	16 (8 M, 8 F)	Randomised, placebo-controlled, double-blind, crossover	24 h (Acute) – 24-h ABPM	150 mg every 8 h (450 mg total/24 h)	24-h ABPM: SBP ↓ 5 mmHg (Dehydra 133 [95% CI 122.9–143.1] vs placebo 138 [95% CI 126.8–149.2]), *p* < 0.001; MAP ↓ 3 mmHg (Dehydra 103 [95% CI 94.5–111.5] vs placebo 106 [95% CI 97.5–114.5]), *p* < 0.001; DBP NS Night-time (sleep): SBP ↓ 7 mmHg (Dehydra 124 [95% CI 114.9–133.1] vs placebo 131 [95% CI 119.8–142.2]), *p* = 0.001; MAP ↓ 4 mmHg (Dehydra 95 [95% CI 88.1–101.9] vs placebo 99 [95% CI 90.5–107.5]), p < 0.05	CBD (*n* = 16): mild drowsiness 2/16 (12.5%), mild diarrhoea 2/16 (12.5%) Placebo (*n* = 16) : mild drowsiness 2/16 (12.5%), mild diarrhoea 1/16 (6.3%)
Dujic et al. (2023)	Treated and Untreated hypertension	69 (40 M, 29 F) *There were 70 participants, one withdrew before first dose and was excluded from all analyses	Randomised, placebo-controlled, triple-blind, crossover	5 weeks (Chronic) – 24-h ABPM at 2.5 & 5 weeks	225–300 mg/day (weeks 0–2.5); 375–450 mg/day (weeks 2.5–5), dosage was weight-based	24‑h ABPM at 2.5 wk (primary): MAP ↓ 3.22 ± 0.90 mmHg (Dehydra) [95% CI − 5.44 to − 1.01], p = 0.002; SBP↓ 4.76 ± 1.24 mmHg (Dehydra) [95% CI − 7.80 to − 1.72], *p* < 0.001; DBP↓ 2.25 ± 0.80 mmHg (Dehydra) [95% CI − 6.01 to − 0.30], *p* = 0.019. No further reduction with uptitration (NS)	CBD (n = 69): diarrhoea 3/69 (4.3%), bloating 2/69 (2.9%), headache 1/69 (1.4%), nausea 1/69 (1.4%), hypersomnia 1/69 (1.4%) Placebo (n = 69): bloating 2/69 (2.9%), headache 2/69 (2.9%), hypersomnia 1/69 (1.4%), constipation 1/69 (1.4%)

^*^M male, *F* female, *SBP* systolic blood pressure, *DBP* diastolic blood pressure, *MAP* mean arterial pressure, *ABPM* ambulatory blood pressure monitoring, *AE* adverse event, *NS* non-significant. Sample (n) refers to participants who have completed the study and had findings analysed per protocol

**Fig. 1:**
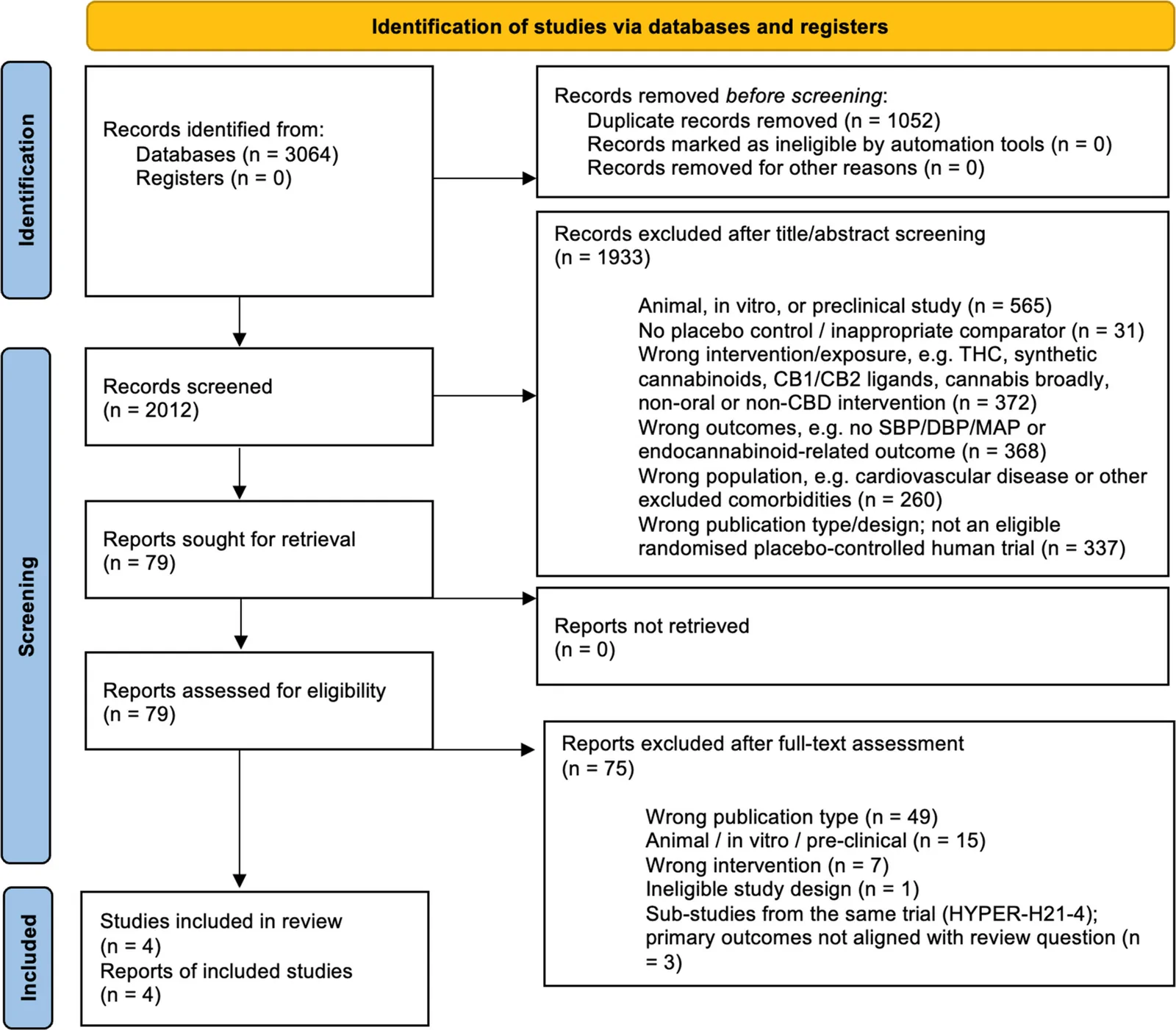
PRISMA flow diagram of study selection

The initial search yielded 3064 articles, of which 2012 titles and abstracts were reviewed, and 4 articles were included in the final analysis, comprising 120 participants. A flow chart of article retrieval and selection is presented in Fig. 1.

The total sample from the final 4 studies included in the review consisted of 120 participants (83 males and 37 females), all adults. While Dragun et al. (2023) and Dujic et al. (2023) focused on individuals diagnosed with various forms of hypertension, ranging from mild to untreated cases, the studies by Jadoon et al. (2017) and Sultan et al. (2020) specifically involved healthy volunteers instead.

## Results

### Primary outcome: dosage effects on blood pressure

The primary outcome across all included studies was the effect of cannabidiol (CBD) on blood pressure (BP), including systolic, diastolic, and mean arterial pressure (MAP). It was observed that CBD dosing led to reductions in BP parameters (Table 2). Across included studies, CBD doses ranged from 225 mg/day to 600 mg/day, administered either as a single acute dose or as repeated daily dosing.

Single-dose administration of 600 mg CBD was evaluated in healthy volunteers by Jadoon et al. (2017) and Sultan et al. (2020). Jadoon et al. reported significant reductions in resting systolic blood pressure (SBP) (− 6 mmHg) and diastolic blood pressure (DBP) (− 5 mmHg, two hours after dosing. Sultan et al. observed a modest reduction in resting mean arterial pressure (MAP) (− 2 mmHg) following acute administration.

Repeated dosing was assessed in three studies. Dragun et al. (2023) administered 150 mg CBD every 8 h over 24 h (total 450 mg), reporting significant reductions in 24-h ambulatory SBP (− 5 mmHg,) and MAP (− 3 mmHg). Sultan et al. (2020) evaluated 600 mg/day over seven days, with no significant changes observed in 24-h ambulatory blood pressure. Dujic et al. (2023) administered weight-based dosing (225–300 mg/day for 2.5 weeks, followed by 375–450 mg/day for 2.5 weeks), reporting significant reductions in ambulatory SBP (- 4.76), DBP (- 2.25), and MAP (- 3.22) at 2.5 weeks, with no additional reductions following dose uptitration.

### Stress and sleep

Stress-evoked blood pressure reductions were consistently observed in healthy volunteers and in at least one chronic phase assessment. In Jadoon et al. (2017), CBD reduced SBP during isometric handgrip (− 6 mmHg), mental arithmetic (− 8 mmHg) and cold pressor (− 8 mmHg), with a concurrent reduction in MAP during cold pressor (− 5 mmHg). In Sultan et al. (2020), CBD reduced stress-associated SBP (− 6 mmHg) with early minute-specific effects. Following 7 days of dosing, Sultan et al. (2020) reported that the resting clinic effect was not maintained, but a stress-associated SBP reduction persisted on day 7 (− 5.7 mmHg). Sleep-related effects were reported in Dragun et al. (2023), where night-time ABPM showed greater reductions than daytime readings, including SBP (− 7 mmHg) and MAP (− 4 mmHg) during sleep.

### Population

In healthy volunteers, CBD administration was associated with reductions in resting clinic blood pressure (Jadoon et al. 2017; Sultan et al. 2020), with systolic blood pressure decreasing by approximately 6 mmHg and diastolic blood pressure by approximately 5 mmHg in Jadoon et al. (2017). These effects were primarily observed following acute or short-term dosing, with limited effects on 24-h ambulatory blood pressure as seen in Sultan et al. (2020).

In contrast, in treated and untreated hypertensive populations (Dragun et al. 2023; Dujic et al. 2023), CBD administration was associated with reductions in ambulatory blood pressure, including 24-h systolic blood pressure reductions of approximately 4–5 mmHg and mean arterial pressure reductions of 3–4 mmHg for both Dragun et al. (2023) and Dujic et al. (2023).

### Formulation

Both standard oral CBD (Jadoon et al. 2017; Sultan et al. 2020) and the Dehydra formulation (Dragun et al. 2023; Dujic et al. 2023) were associated with reductions in blood pressure. Standard oral CBD in Jadoon et al. (2017) reduced resting systolic blood pressure in healthy volunteers by 6 mmHg and Sultan et al. (2020) showed reduction in resting mean arterial pressure by 2 mmHg. The Dehydra formulation showed reductions of 5 mmHg for systolic blood pressure and 3 mmHg for mean arterial pressure in Dragun et al. (2023). Dujic et al. (2023) also showed similar reductions with Dehydra formulation as seen in Table 2. No direct formulation comparisons were conducted.

### Side effects

CBD was generally well tolerated, with adverse events largely mild. No adverse events were reported in Jadoon et al. (2017). In Sultan et al. (2020), mild symptoms occurred at low frequency in the CBD arm (lack of appetite, headache, insomnia, hyperactivity, dysuria), with similarly infrequent placebo events (migraine and light-headedness). In Dragun et al. (2023), mild drowsiness and mild diarrhoea were each reported by participants in the CBD condition, with comparable drowsiness in placebo and slightly fewer diarrhoea events. In Dujic et al. (2023), adverse events were uncommon and mainly gastrointestinal (diarrhoea, bloating), with headache, nausea and hypersomnia; placebo events were similarly low frequency.

Details from all four studies, including participant characteristics, study design, CBD dose and administration route, BP outcomes, and side effects, are summarised in Table 2 (see Master Table of Study Characteristics and Results).

**Fig. 2:**
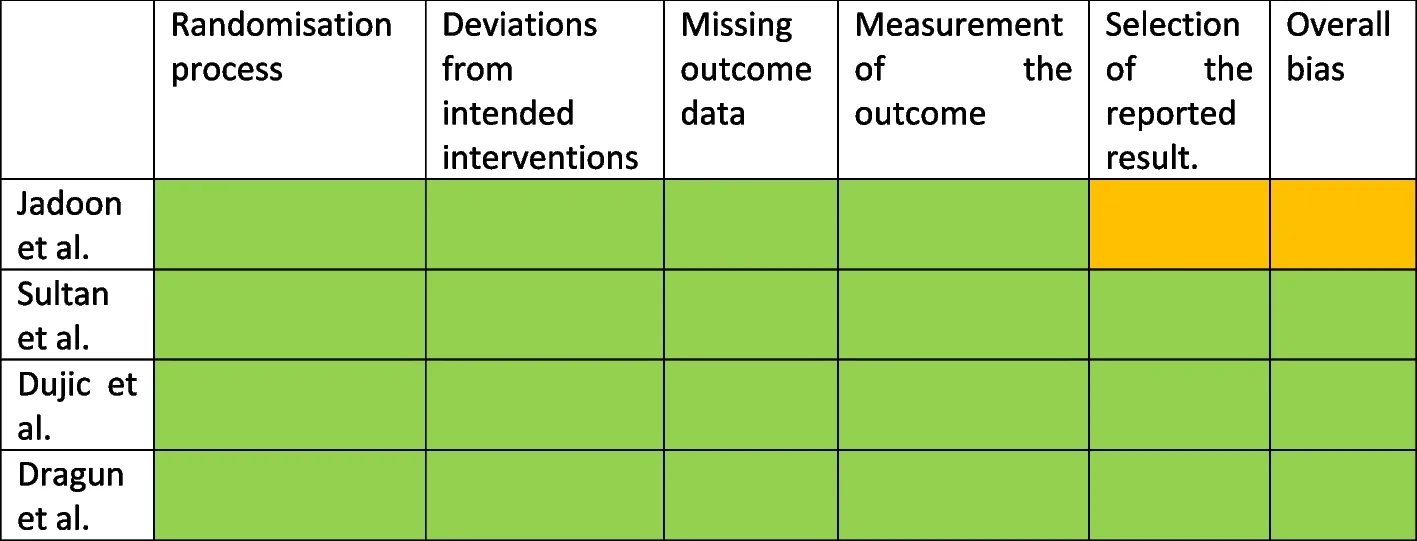
Cochrane Risk of Bias 2 (RoB2) assessment for the included studies. Colour coding indicates the level of bias: Green = Low risk; Orange = Some concerns; Red = High risk

Most of the included studies demonstrated low risk across all five domains (Fig. 2). Jadoon et al. (2017) was judged to have “some concerns” in the domain of “selection of the reported result” due to unclear pre-specification of analysis plans, leaving some uncertainty regarding selective reporting (Supplement 2). No study was assessed as having a high risk of bias in any domain. These findings support the overall methodological robustness of the included trials, though cautious interpretation is warranted where reporting transparency was limited. Both AR and SF were in general agreement with the evaluation of bias.

## Discussion

This systematic review assesed the evidence of the effects of oral cannabidiol (CBD) supplementation on blood pressure (BP) regulation exclusively on human randomised controlled trials (RCTs). The aim of this systematic review was to assess the safety and efficacy of CBD in reducing BP among individuals with and without hypertension. Observational data indicate that hypertension is already cited as a reason for cannabis use. While not specific to CBD and not indicative of efficacy, this suggests a pre-existing demand for cannabinoid-based blood pressure management (Hakkarainen et al. 2015) and provides context for the clinical findings reported here. Across the included studies, the most pronounced BP reductions were observed following acute CBD administration at doses of 600 mg per day (Jadoon et al. 2017; Sultan et al. 2020).

A recent systematic review and meta-analysis included both normotensive and hypertensive participants and quantitatively pooled acute and chronic blood pressure outcomes (Candeloro et al. 2025). In contrast, the present review provides a structured narrative synthesis across resting, stress-related, ambulatory, and sleep conditions, as well as formulation-specific and safety outcomes, reflecting substantial clinical heterogeneity.

### Dosage

Acute administration of moderate-to-high (150–600 mg) CBD doses produced notable reductions in BP. Jadoon et al. (2017) and Dragun et al. (2023) each observed systolic BP decreases exceeding 5 mmHg, a clinically meaningful threshold, as a 5 mmHg systolic reduction is associated with a roughly 10% lower risk of major cardiovascular events. Similarly, Sultan et al. (2020) demonstrated systolic reductions > 5 mmHg with 600 mg, extending benefits to stress-induced BP responses. By contrast, Dujic et al. (2023) showed that chronic titration from 225–300 mg/day significantly lowered systolic, diastolic, and MAP at 2.5 weeks, but further escalation to 375–450 mg/day did not enhance effects. This indicates a plateau in the blood pressure–lowering efficacy of CBD, whereby higher doses did not confer incremental cardiovascular benefit beyond the moderate-dose range. Collectively, these findings indicate that CBD was observed to reduce systolic and mean arterial pressure, while effects on diastolic blood pressure were less consistent across studies; acute dosing appeared effective, whereas higher chronic doses did not confer additional benefit.

Across trials, a dose–response relationship between CBD dosage and blood pressure reduction was observed. Acute doses in the moderate-to-high range were consistently associated with clinically meaningful reductions in systolic BP and mean arterial pressure (Canoy et al. 2022). Notably, lower or titrated doses required longer exposure to achieve comparable effects, while diastolic BP responses remained less consistent across dose ranges. Collectively, these findings suggest that CBD exhibits varied blood pressure reduction than a simple linear dose–response relationship, with diminishing returns at higher chronic doses.

### Stress and sleep

Stress is associated with hypertension, including psychosocial stress which has been often included as a non-modifiable risk factor (Munakata 2018). Stress paradigms used in both Jadoon et al. (2017) and Sultan et al. (2020) included the isometric handgrip test, which induces cardiovascular stress via sustained muscle contraction. Jadoon et al. further employed the cold pressor test (hand immersion in cold water) and a mental arithmetic task designed to elicit cognitive stress through time-pressured calculations. These primarily capture acute sympathetic activation (Jatoi et al. 2014), and only partially reflect the complexity of chronic psychosocial stress (Giles et al. 2014). The most consistent effects of CBD have been observed within these controlled stress settings. Both Jadoon et al. (2017) and Sultan et al. (2020) found that a single 600 mg dose blunted stress-induced systolic BP rises by ~ 5–6 mmHg, with Sultan further showing this effect persisted after 7 days despite resting values returning to baseline. Therefore, the consistent attenuation of BP surges under these stress conditions supports a sympatholytic action of CBD, although extrapolation to real-world psychosocial stress requires caution.

Complementing these results, Dragun et al. (2023) observed greater reductions during sleep, suggesting CBD’s effects may vary with circadian state. This is consistent with evidence that nocturnal dipping is a key component of cardiovascular health and is closely linked to emotional regulation and stress-related processes (Casagrande et al. 2020).

### Population

Healthy volunteers (Jadoon et al. 2017; Sultan et al. 2020) demonstrated greater reductions in blood pressure compared with hypertensive patients (Dragun et al. 2023; Dujic et al. 2023). This difference may be partly explained by the higher doses administered to the healthy cohorts (Sultan et al. 2020; Dujic et al. 2023), which complicates direct comparison with trials in healthy individuals. In particular, hypertensive patients often exhibit vascular remodelling, arterial stiffness, endothelial dysfunction (Humphrey 2021). Therefore, physiological differences may influence the blood pressure–lowering effects of CBD. Thus, dose-matched studies are needed to determine how CBD influences BP in hypertensive and normotensive states.

### Formulation

An important methodological difference across trials was the formulation of CBD administered. Findings highlight the importance of formulation in interpreting CBD’s cardiovascular effects and suggest optimised delivery systems such as DehydraTECH™ oral capsules, a lipid-based delivery system designed to enhance absorption and reduce phytochemical degradation during first pass metabolism (Patrician et al. 2019) could achieve clinically relevant BP lowering effects compared to conventional purified CBD capsules (Jadoon et al. 2017; Sultan et al. 2020).

### Side effects

In terms of safety, CBD was generally well tolerated across all included studies, with no serious adverse cardiovascular events reported. Side effects, particularly at higher doses or with longer use, included insomnia, and headaches (Sultan et al. 2020). Sultan et al. (2020) reported a wider range of adverse events such as hyperactivity and dysuria. Severity of most symptoms were mild to moderate and transient, underscoring the importance of dose titration and monitoring, especially in clinical populations. Notably, the absence of cardiovascular-specific adverse effects supports the short-term safety of CBD in individuals with elevated BP. However, medical cannabis products require dedicated monitoring frameworks, given their classification as herbal therapies and the inherent variability in composition, formulation, and bioavailability (Fortin and Massin 2020).

### Limitations

Firstly, the evidence base is limited to only four randomised controlled trials included, highlighting the emerging area of CBD’s anti-hypertensive properties. The small number of studies restricts the ability to perform robust meta-analyses or identify definitive efficacy patterns. Additionally, substantial heterogeneity was present across these studies, arising from variations in participant health status, CBD dosages, sample size, methods of administration, trial length, outcome measurements and measurements timepoints. This variability significantly complicates direct comparison and interpretation of results. Furthermore, as all included studies evaluated cannabidiol administered via oral ingestion, the findings cannot be generalised to alternative routes of administration that may differ in bioavailability and onset of action. Finally, publication bias remains a concern, particularly given that several included trials were industry-sponsored with those evaluating the Dehydra formulation.

This review has some methodological limitations. Restricting to English-language studies may have introduced language bias, and searching only four databases without grey literature or trial registries increases the risk of missing unpublished work. Screening and data extraction relied on consensus, but inter-rater reliability was not formally tested.

### Future directions

High heterogeneity across existing studies highlights the need for standardised dosing protocols and more robust clinical trials. Future research should clarify dose response relationships to better characterise CBD’s antihypertensive effects and inform optimal dosing. As current evidence largely excludes resistant hypertension, studies should assess whether CBD provides adjunctive benefits in this population. Longitudinal studies of at least 12 months are needed to determine whether blood pressure–lowering effects are sustained over time, with longer follow-up required to assess attenuation and the need for dose adjustments (Canoy et al. 2022). Finally, alternative methods of administration beyond standard oral capsules such as sublingual, intranasal, inhalation and topical application routes should be explored, as these may enhance bioavailability and therapeutic potential (O’Sullivan et al. 2024). Although CBD is generally well tolerated, a recent randomised study in healthy adults reported elevations in liver transaminases exceeding three times the upper limit of normal in a subset of participants receiving CBD, with no such elevations observed in the placebo group. These changes were largely reversible following discontinuation, although the long-term clinical significance remains unclear (Florian et al. 2025). While liver function tests were infrequently used in the studies included in the present systematic evaluation, these recent findings highlight the need for careful safety monitoring.

In parallel, population-based surveys assessing motivations for CBD consumption should explicitly include hypertension as a potential therapeutic indication. This would help identify individuals who may derive benefit from CBD as a single therapeutic agent rather than through multiple concurrent medications whose combined efficacy and side-effect profiles have not yet been systematically evaluated. At the same time, systematic evaluation of CBD’s safety profile remains essential, particularly with respect to potential drug–drug interactions. Further investigation is warranted to assess the efficacy and safety of CBD in combination with commonly prescribed antihypertensive medication classes and given CBD’s known interactions with drugs metabolised via CYP450 pathways (Smith and Gruber 2023), careful monitoring for interactions with beta-blockers, calcium channel blockers, and anticoagulants is advised. Future studies must therefore explicitly address safety outcomes and interaction potential to support comprehensive, evidence-based clinical guidance for healthcare professionals.

## Conclusion

This review of four human randomised controlled trials suggests that CBD may lower systolic, diastolic, and mean arterial pressure, with the most consistent effects seen during stress and at high acute doses. CBD was generally well tolerated, with only mild adverse effects reported, and no cardiovascular safety concerns identified. Nonetheless, the evidence base is small, heterogeneous in design, and largely limited to healthy or mildly hypertensive populations, with little data on resistant hypertension or long-term use. Accordingly, CBD cannot yet be recommended as an antihypertensive treatment based on the existing clinical evidence, rather than due to a definitive lack of therapeutic potential. Larger, dose-controlled, and longer-duration trials across diverse patient groups with real-world monitoring and patient-reported outcomes may support cautious use. These are needed to establish efficacy, optimal dosing, and clinical applicability. Aside from oral administration, future studies should also evaluate alternative routes such as inhalation or sublingual dosing, which may differ in bioavailability.

## Supplementary Information


Supplementary Material 1.
Supplementary Material 2.


## Data Availability

No datasets were generated or analysed during the current study.

## Abbreviations

AE: Adverse Event
ABPM: Ambulatory Blood Pressure Monitoring
BP: Blood Pressure
CBD: Cannabidiol
CVD: Cardiovascular Disease
CYP450: Cytochrome P450 (enzyme system)
DALY: Disability-Adjusted Life Year
DBP: Diastolic Blood Pressure
F: Female
MAP: Mean Arterial Pressure
M: Male
NS: Non-significant
OTC: Over the Counter
RCT: Randomised Controlled Trial
RoB2: Risk of Bias 2 (Cochrane tool)
SBP: Systolic Blood Pressure
THC: Δ9-Tetrahydrocannabinol
WHO: World Health Organization
