# Supplementary material for: The effects of oral cannabidiol supplementation on blood pressure in adults: a systematic review of randomised controlled trials
Source: J Cannabis Res. 2026 Jun 19;8:96. doi: 10.1186/s42238-026-00463-3 (PMC13531894; doi:10.1186/s42238-026-00463-3)
Supplement: Supplementary file 1 [file 42238_2026_463_MOESM1_ESM.docx]

Supplement 1

# Search strategy – Boolean command

PubMed (via NCBI)

("Cannabidiol"[All Fields] OR "CBD"[All Fields] OR "Cannabinoids"[All Fields]) AND ("Hypertension"[All Fields] OR "Normotension"[All Fields] OR ("High Blood Pressure"[All Fields] OR "Normal Blood Pressure"[All Fields]

**Filters Applied:**

- Language: English
- Design: RCT
- Date range: Inception to September 2024

Web of Science (via Clarivate)

TS=("Cannabidiol" OR "CBD" OR "Cannabinoids")

AND

TS=("Hypertension" OR "Normotension" OR "Normal Blood Pressure" OR “High Blood Pressure”)

**Filters Applied:**

- Language: English
- Design: RCT
- Date range: Inception to September 2024

Scopus (via Elsevier)

TITLE-ABS-KEY("Cannabidiol" OR "CBD" OR "Cannabinoids")

AND

TITLE-ABS-KEY("Hypertension" OR "Normotension" OR "Normal Blood Pressure" OR “High Blood Pressure”)

**Filters Applied:**

- Language: English
- Design: RCT
- Date range: Inception to September 2024

Medline (via OVID)

("Cannabidiol" OR "CBD" OR "Cannabinoids")

AND

("Hypertension" OR "Normotension" OR "Normal Blood Pressure" OR “High Blood Pressure”)

**Filters Applied:**

- Language: English
- Species: Humans
- Date range: Inception to September 2024

# Reports sort for retrieval

| **Title (Accept in Green Fill; Substudies of the HYPER-H21-4 Trial were excluded in Yellow Fill*) Multiple reports originated from the same clinical trial (HYPER-H21-4). There was also a mismatch in placebo-reporting. *** | **Citation** |
| --- | --- |
| A new face of endocannabinoids in pharmacotherapy. Part II: role of endocannabinoids in inflammation-derived cardiovaascular diseases | Zubrzycki M, Liebold A, Janecka A, Zubrzycka M. A new face of endocannabinoids in pharmacotherapy. Part II: role of endocannabinoids in inflammation-derived cardiovaascular diseases. J Physiol Pharmacol. 2014 Apr;65(2):183-91. PMID: 24781728. |
| A single dose of cannabidiol reduces blood pressure in healthy volunteers in a randomized crossover study | Jadoon KA, Tan GD, O'Sullivan SE. A single dose of cannabidiol reduces blood pressure in healthy volunteers in a randomized crossover study. JCI Insight. 2017 Jun 15;2(12):e93760. doi: 10.1172/jci.insight.93760. PMID: 28614793; PMCID: PMC5470879. |
| A Systematic Review of Cannabidiol Dosing in Clinical Populations. | Millar SA, Stone NL, Bellman ZD, Yates AS, England TJ, O'Sullivan SE. A systematic review of cannabidiol dosing in clinical populations. Br J Clin Pharmacol. 2019 Sep;85(9):1888-1900. doi: 10.1111/bcp.14038. Epub 2019 Jul 19. PMID: 31222854; PMCID: PMC6710502. |
| Activation of Cannabinoid CB2 Receptor Ameliorates Atherosclerosis Associated With Suppression of Adhesion Molecules | Zhao Y, Yuan Z, Liu Y, Xue J, Tian Y, Liu W, Zhang W, Shen Y, Xu W, Liang X, Chen T. Activation of cannabinoid CB2 receptor ameliorates atherosclerosis associated with suppression of adhesion molecules. J Cardiovasc Pharmacol. 2010 Mar;55(3):292-8. doi: 10.1097/FJC.0b013e3181d2644d. PMID: 20075743. |
| Acute and Long-Term Effects of Cannabinoids on Hypertension and Kidney Injury. | Golosova, D., Levchenko, V., Kravtsova, O. et al. Acute and long-term effects of cannabinoids on hypertension and kidney injury. Sci Rep 12, 6080 (2022). <https://doi.org/10.1038/s41598-022-09902-6> |
| An Overview on Plants Cannabinoids Endorsed with Cardiovascular Effects. | Dimmito MP, Stefanucci A, Della Valle A, Scioli G, Cichelli A, Mollica A. An overview on plants cannabinoids endorsed with cardiovascular effects. Biomed Pharmacother. 2021 Oct;142:111963. doi: 10.1016/j.biopha.2021.111963. Epub 2021 Jul 28. PMID: 34332376. |
| Antihypertensive Effects of CBD Are Mediated by Altered Inflammatory Response: A Sub-Study of the HYPER-H21-4 Trial. | Urlic, H., Kumric, M., Dujic, G., Vrdoljak, J., Supe-Domic, D., Dujic, Z. and Bozic, J. (2023). Antihypertensive Effects of CBD Are Mediated by Altered Inflammatory response: a sub-study of the HYPER-H21-4 Trial. Journal of Functional Foods, [online] 110, p.105873. doi:https://doi.org/10.1016/j.jff.2023.105873. |
| Application of medical cannabis in unstable angina and coronary artery disease: A case report. | Shaffer BL, Davis GM, Incitti MA, Piper BJ, Entler BV. Application of medical cannabis in unstable angina and coronary artery disease: A case report. Medicine (Baltimore). 2021 Mar 19;100(11):e25172. doi: 10.1097/MD.0000000000025172. PMID: 33726006; PMCID: PMC7982176. |
| At the Heart of Microbial Conversations: Endocannabinoids and the Microbiome in Cardiometabolic Risk. | Khan RN, Maner-Smith K, A Owens J, Barbian ME, M Jones R, R Naudin C. At the heart of microbial conversations: endocannabinoids and the microbiome in cardiometabolic risk. Gut Microbes. 2021 Jan-Dec;13(1):1-21. doi: 10.1080/19490976.2021.1911572. PMID: 33896380; PMCID: PMC8078674. |
| Cannabidiol treatment improves metabolic profile and decreases hypothalamic inflammation caused by maternal obesity | Rodrigues FDS, Jantsch J, Fraga GF, Dias VS, Eller S, De Oliveira TF, Giovenardi M, Guedes RP. Cannabidiol treatment improves metabolic profile and decreases hypothalamic inflammation caused by maternal obesity. Front Nutr. 2023 Mar 9;10:1150189. doi: 10.3389/fnut.2023.1150189. PMID: 36969815; PMCID: PMC10033544. |
| Cannabidiol-Loaded Nanocarriers and Their Therapeutic Applications | Assadpour, E.; Rezaei, A.; Das, S.S.; Krishna Rao, B.V.; Singh, S.K.; Kharazmi, M.S.; Jha, N.K.; Jha, S.K.; Prieto, M.A.; Jafari, S.M. Cannabidiol-Loaded Nanocarriers and Their Therapeutic Applications. Pharmaceuticals 2023, 16, 487. <https://doi.org/10.3390/ph16040487> |
| Cannabinoid 1 Receptor Blockade Reduces Atherosclerosis with Enhances Reverse Cholesterol Transport | Sugamura K, Sugiyama S, Fujiwara Y, Matsubara J, Akiyama E, Maeda H, Ohba K, Matsuzawa Y, Konishi M, Nozaki T, Horibata Y, Kaikita K, Sumida H, Takeya M, Ogawa H. Cannabinoid 1 receptor blockade reduces atherosclerosis with enhances reverse cholesterol transport. J Atheroscler Thromb. 2010 Feb 26;17(2):141-7. doi: 10.5551/jat.2865. Epub 2010 Feb 3. PMID: 20124735. |
| Cannabinoid and GABA Modulation of Sympathetic Nerve Activity and Blood Pressure in the Dorsal Periaqueductal Gray of the Rat. | Dean C. Cannabinoid and GABA modulation of sympathetic nerve activity and blood pressure in the dorsal periaqueductal gray of the rat. Am J Physiol Regul Integr Comp Physiol. 2011 Dec;301(6):R1765-72. doi: 10.1152/ajpregu.00398.2011. Epub 2011 Sep 21. PMID: 21940402. |
| Cannabinoid CB1 Receptor Antagonists for Atherosclerosis and Cardiometabolic Disorders New Hopes, Old Concerns? | Pacher P. Cannabinoid CB1 receptor antagonists for atherosclerosis and cardiometabolic disorders: new hopes, old concerns? Arterioscler Thromb Vasc Biol. 2009 Jan;29(1):7-9. doi: 10.1161/ATVBAHA.108.178129. PMID: 19092136; PMCID: PMC2669438. |
| Cannabinoid Receptor Type 2 Activation in Atherosclerosis and Acute Cardiovascular Diseases | Carbone F, Mach F, Vuilleumier N, Montecucco F. Cannabinoid receptor type 2 activation in atherosclerosis and acute cardiovascular diseases. Curr Med Chem. 2014;21(35):4046-58. doi: 10.2174/0929867321666140915141332. PMID: 25245379. |
| Cannabinoid receptors in acute and chronic complications of atherosclerosis | Mach F, Montecucco F, Steffens S. Cannabinoid receptors in acute and chronic complications of atherosclerosis. Br J Pharmacol. 2008 Jan;153(2):290-8. doi: 10.1038/sj.bjp.0707517. Epub 2007 Oct 29. PMID: 17965744; PMCID: PMC2219535. |
| Cannabinoid receptors in atherosclerosis. | Steffens S, Mach F. Cannabinoid receptors in atherosclerosis. Curr Opin Lipidol. 2006 Oct;17(5):519-26. doi: 10.1097/01.mol.0000245257.17764.b2. PMID: 16960500. |
| Cannabinoid-sensitive receptors in cardiac physiology and ischaemia | Puhl SL. Cannabinoid-sensitive receptors in cardiac physiology and ischaemia. Biochim Biophys Acta Mol Cell Res. 2020 Mar;1867(3):118462. doi: 10.1016/j.bbamcr.2019.03.009. Epub 2019 Mar 16. PMID: 30890410. |
| Cannabinoids and atherosclerosis. | Fisar Z. Cannabinoids and atherosclerosis. Prague Med Rep. 2009;110(1):5-12. PMID: 19591373. |
| Cannabinoids and Atherosclerotic Coronary Heart Disease | Singla S, Sachdeva R, Mehta JL. Cannabinoids and atherosclerotic coronary heart disease. Clin Cardiol. 2012 Jun;35(6):329-35. doi: 10.1002/clc.21962. Epub 2012 Jan 25. PMID: 22278660; PMCID: PMC6652534. |
| Cannabinoids and cardiovascular disease: The outlook for clinical treatments | Ashton JC, Smith PF. Cannabinoids and cardiovascular disease: the outlook for clinical treatments. Curr Vasc Pharmacol. 2007 Jul;5(3):175-85. doi: 10.2174/157016107781024109. PMID: 17627561. |
| Cannabinoids and Cardiovascular System. | Bondarenko AI. Cannabinoids and Cardiovascular System. Adv Exp Med Biol. 2019;1162:63-87. doi: 10.1007/978-3-030-21737-2_5. PMID: 31332735. |
| Cannabinoids and Endocannabinoids in Metabolic Disorders with Focus on Diabetes | Di Marzo V, Piscitelli F, Mechoulam R. Cannabinoids and endocannabinoids in metabolic disorders with focus on diabetes. Handb Exp Pharmacol. 2011;(203):75-104. doi: 10.1007/978-3-642-17214-4_4. PMID: 21484568. |
| Cannabinoids as therapeutic agents in cardiovascular disease:: a tale of passions and illusions | Mendizábal VE, Adler-Graschinsky E. Cannabinoids as therapeutic agents in cardiovascular disease: a tale of passions and illusions. Br J Pharmacol. 2007 Jun;151(4):427-40. doi: 10.1038/sj.bjp.0707261. Epub 2007 Apr 23. PMID: 17450170; PMCID: PMC2013961. |
| Cannabinoids for Treating Cardiovascular Disorders: Putting Together a Complex Puzzle. | Eid BG. Cannabinoids for Treating Cardiovascular Disorders: Putting Together a Complex Puzzle. J Microsc Ultrastruct. 2018 Oct-Dec;6(4):171-176. doi: 10.4103/JMAU.JMAU_42_18. PMID: 30464888; PMCID: PMC6206749. |
| Cannabinoids in Arterial, Pulmonary, and Portal Hypertension - Mechanisms of Action and Potential Therapeutic Significance. | Malinowska B, Toczek M, Pędzińska-Betiuk A, Schlicker E. Cannabinoids in arterial, pulmonary and portal hypertension - mechanisms of action and potential therapeutic significance. Br J Pharmacol. 2019 May;176(10):1395-1411. doi: 10.1111/bph.14168. Epub 2018 Apr 14. PMID: 29455452; PMCID: PMC6487561. |
| Cannabinoids in the Cardiovascular System. | Ho WSV, Kelly MEM. Cannabinoids in the Cardiovascular System. Adv Pharmacol. 2017;80:329-366. doi: 10.1016/bs.apha.2017.05.002. Epub 2017 Jul 3. PMID: 28826540. |
| Cannabinoids-A New Perspective in Adjuvant Therapy for Pulmonary Hypertension. | Krzyżewska A, Baranowska-Kuczko M, Mińczuk K, Kozłowska H. Cannabinoids-A New Perspective in Adjuvant Therapy for Pulmonary Hypertension. Int J Mol Sci. 2021 Sep 17;22(18):10048. doi: 10.3390/ijms221810048. PMID: 34576212; PMCID: PMC8472313. |
| Cannabis and cannabinoids as an alternative remedy in metabolic syndrome | Claudia Ali Montoya-Alatriste and Francisco Javier Alarcón-Aguilar (2022). Cannabis and cannabinoids as an alternative remedy in metabolic syndrome. Brazilian Journal of Pharmaceutical Sciences, 58. doi:https://doi.org/10.1590/s2175-97902022e20161. |
| Cardiovascular Effects of Cannabinoids. | Wang A, Dey S, Subhan S, Patel J, Frishman WH, Aronow WS. Cardiovascular Effects of Cannabinoids. Cardiol Rev. 2025 Jan-Feb 01;33(1):36-40. doi: 10.1097/CRD.0000000000000566. Epub 2023 Jun 29. PMID: 37382432. |
| Cardiovascular Effects of Prolonged Delta-9-Tetrahydrocannabinol Ingestion. | Benowitz NL, Jones RT. Cardiovascular effects of prolonged delta-9-tetrahydrocannabinol ingestion. Clin Pharmacol Ther. 1975 Sep;18(3):287-97. doi: 10.1002/cpt1975183287. PMID: 1164818. |
| Cardiovascular Side Effects Related to the Use of Synthetic Cannabinoids 'Bonzai': Two Case Reports. | Atik SU, Dedeoğlu R, Varol F, Çam H, Eroğlu AG, Saltık L. Cardiovascular side effects related with use of synthetic cannabinoids "bonzai" : two case reports. Turk Pediatri Ars. 2015 Mar 1;50(1):61-4. doi: 10.5152/tpa.2015.2609. PMID: 26078698; PMCID: PMC4462325. |
| CBD supplementation reduces arterial blood pressure via modulation of the sympatho-chromaffin system: A substudy from the HYPER-H21-4 trial | Kumric M, Dujic G, Vrdoljak J, Svagusa K, Kurir TT, Supe-Domic D, Dujic Z, Bozic J. CBD supplementation reduces arterial blood pressure via modulation of the sympatho-chromaffin system: A substudy from the HYPER-H21-4 trial. Biomed Pharmacother. 2023 Apr;160:114387. doi: 10.1016/j.biopha.2023.114387. Epub 2023 Feb 11. PMID: 36780785 |
| Chronic Effects of Oral Cannabidiol Delivery on 24-h Ambulatory Blood Pressure in Patients with Hypertension (HYPER-H21-4): A Randomized, Placebo-Controlled, and Crossover Study. | Dujic G, Kumric M, Vrdoljak J, Dujic Z, Bozic J. Chronic Effects of Oral Cannabidiol Delivery on 24-h Ambulatory Blood Pressure in Patients with Hypertension (HYPER-H21-4): A Randomized, Placebo-Controlled, and Crossover Study. Cannabis Cannabinoid Res. 2024 Aug;9(4):979-989. doi: 10.1089/can.2022.0320. Epub 2023 Apr 21. PMID: 37093160. |
| Circulating endocannabinoid levels, abdominal adiposity and related cardiometabolic risk factors in obese men | Côté M, Matias I, Lemieux I, Petrosino S, Alméras N, Després JP, Di Marzo V. Circulating endocannabinoid levels, abdominal adiposity and related cardiometabolic risk factors in obese men. Int J Obes (Lond). 2007 Apr;31(4):692-9. doi: 10.1038/sj.ijo.0803539. Epub 2007 Jan 16. PMID: 17224929. |
| Differential Interaction of Cannabidiol with Biomembranes Dependent on Cholesterol Concentration | Perez E, Ceja-Vega J, Krmic M, Gamez Hernandez A, Gudyka J, Porteus R, Lee S. Differential Interaction of Cannabidiol with Biomembranes Dependent on Cholesterol Concentration. ACS Chem Neurosci. 2022 Apr 6;13(7):1046-1054. doi: 10.1021/acschemneuro.2c00040. Epub 2022 Mar 17. PMID: 35298887. |
| Drugs of abuse and cardiovascular risk: the cannabinoids | Abrignani MG, Ciccirillo F, Temporelli PL, Cesaro A, Binaghi G, Maloberti A, Cappelletto C, Oliva F, Riccio C, Caldarola P, Gabrielli D, Colivicchi F. Sostanze d’abuso e rischio cardiovascolare: i cannabinoidi [Substances of abuse and cardiovascular risk: cannabinoids]. G Ital Cardiol (Rome). 2023 Jun;24(6):455-465. Italian. doi: 10.1714/4041.40205. PMID: 37227205. |
| Effects of CBD Supplementation on Ambulatory Blood Pressure and Serum Urotensin-II Concentrations in Caucasian Patients with Essential Hypertension: A Sub-Analysis of the HYPER-H21-4 Trial. | Kumric M, Dujic G, Vrdoljak J, Supe-Domic D, Bilopavlovic N, Dolic K, Dujic Z, Bozic J. Effects of CBD supplementation on ambulatory blood pressure and serum urotensin-II concentrations in Caucasian patients with essential hypertension: A sub-analysis of the HYPER-H21-4 trial. Biomed Pharmacother. 2023 Aug;164:115016. doi: 10.1016/j.biopha.2023.115016. Epub 2023 Jun 13. PMID: 37321059. |
| Effects of Hypertension on Vasorelaxation to Endocannabinoids In Vitro. | Wheal AJ, Randall MD. Effects of hypertension on vasorelaxation to endocannabinoids in vitro. Eur J Pharmacol. 2009 Jan 28;603(1-3):79-85. doi: 10.1016/j.ejphar.2008.11.061. Epub 2008 Dec 9. PMID: 19100733. |
| Effects of Tetrahydrocannabinol on Arterial and Intraocular Hypertension. | Crawford WJ, Merritt JC. Effects of tetrahydrocannabinol on arterial and intraocular hypertension. Int J Clin Pharmacol Biopharm. 1979 May;17(5):191-6. PMID: 468444. |
| Endocannabinoid Signalling in Atherosclerosis and Related Metabolic Complications | Guillamat-Prats R, Rami M, Herzig S, Steffens S. Endocannabinoid Signalling in Atherosclerosis and Related Metabolic Complications. Thromb Haemost. 2019 Apr;119(4):567-575. doi: 10.1055/s-0039-1678738. Epub 2019 Feb 15. PMID: 30769363. |
| Endocannabinoid system and its implications for obesity and cardiometabolic risk | Saavedra LE. Endocannabinoid system and cardiometabolic risk. Clin Pharmacol Ther. 2007 Nov;82(5):591-4. doi: 10.1038/sj.clpt.6100373. Epub 2007 Sep 26. PMID: 17898706. |
| Endocannabinoid System as a Potential Mechanism for n-3 Long-Chain Polyunsaturated Fatty Acid Mediated Cardiovascular Protection. | Wainwright CL, Michel L. Endocannabinoid system as a potential mechanism for n-3 long-chain polyunsaturated fatty acid mediated cardiovascular protection. Proc Nutr Soc. 2013 Nov;72(4):460-9. doi: 10.1017/S0029665113003406. Epub 2013 Sep 11. PMID: 24020800. |
| Endocannabinoid system in cardiovascular disorders - new pharmacotherapeutic opportunities. | Cunha P, Romão AM, Mascarenhas-Melo F, Teixeira HM, Reis F. Endocannabinoid system in cardiovascular disorders - new pharmacotherapeutic opportunities. J Pharm Bioallied Sci. 2011 Jul;3(3):350-60. doi: 10.4103/0975-7406.84435. PMID: 21966155; PMCID: PMC3178941. |
| Endocannabinoids Acting at Cannabinoid-1 Receptors Regulate Cardiovascular Function in Hypertension. | Bátkai S, Pacher P, Osei-Hyiaman D, Radaeva S, Liu J, Harvey-White J, Offertáler L, Mackie K, Rudd MA, Bukoski RD, Kunos G. Endocannabinoids acting at cannabinoid-1 receptors regulate cardiovascular function in hypertension. Circulation. 2004 Oct 5;110(14):1996-2002. doi: 10.1161/01.CIR.0000143230.23252.D2. Epub 2004 Sep 27. PMID: 15451779; PMCID: PMC2756479. |
| Endocannabinoids and Cardiac Contractile Function: Pathophysiological Implications. | Bátkai S, Pacher P. Endocannabinoids and cardiac contractile function: pathophysiological implications. Pharmacol Res. 2009 Aug;60(2):99-106. doi: 10.1016/j.phrs.2009.04.003. PMID: 19569260; PMCID: PMC2768548. |
| Endocannabinoids and cardiovascular prevention: real progress? | Nodari S, Manerba A, Metra M, Dei Cas L. Endocannabinoids and cardiovascular prevention: real progress? Heart Int. 2007;3(1):27. doi: 10.4081/hi.2007.27. Epub 2007 Jun 15. PMID: 21977272; PMCID: PMC3184683. |
| Endocannabinoids and the heart. | Hiley CR. Endocannabinoids and the heart. J Cardiovasc Pharmacol. 2009 Apr;53(4):267-76. doi: 10.1097/FJC.0b013e318192671d. PMID: 19276990; PMCID: PMC2728560. |
| Endocannabinoids, Blood Pressure, and the Human Heart - Sarzani, R (May 2008). | Sarzani R. Endocannabinoids, blood pressure and the human heart. J Neuroendocrinol. 2008 May;20 Suppl 1:58-62. doi: 10.1111/j.1365-2826.2008.01677.x. PMID: 18426501. |
| Enhanced Vasorelaxant Effects of the Endocannabinoid-Like Mediator, Oleamide, in Hypertension. | Hopps JJ, Dunn WR, Randall MD. Enhanced vasorelaxant effects of the endocannabinoid-like mediator, oleamide, in hypertension. Eur J Pharmacol. 2012 Jun 5;684(1-3):102-7. doi: 10.1016/j.ejphar.2012.03.027. Epub 2012 Mar 23. PMID: 22465182. |
| High-Dose Cannabidiol Induced Hypotension after Global Hypoxia-Ischemia in Piglets. | Garberg HT, Solberg R, Barlinn J, Martinez-Orgado J, Løberg EM, Saugstad OD. High-Dose Cannabidiol Induced Hypotension after Global Hypoxia-Ischemia in Piglets. Neonatology. 2017;112(2):143-149. doi: 10.1159/000471786. Epub 2017 Jun 1. PMID: 28564654. |
| How do phytocannabinoids affect cardiovascular health? An update on the most common cardiovascular diseases. | Dziemitko S, Harasim-Symbor E, Chabowski A. How do phytocannabinoids affect cardiovascular health? An update on the most common cardiovascular diseases. Ther Adv Chronic Dis. 2023 Jan 6;14:20406223221143239. doi: 10.1177/20406223221143239. PMID: 36636553; PMCID: PMC9830002. |
| Multiomic Analysis Reveals Disruption of Cholesterol Homeostasis by Cannabidiol in Human Cell Lines | Guard SE, Chapnick DA, Poss ZC, Ebmeier CC, Jacobsen J, Nemkov T, Ball KA, Webb KJ, Simpson HL, Coleman S, Bunker E, Ramirez A, Reisz JA, Sievers R, Stowell MHB, D'Alessandro A, Liu X, Old WM. Multiomic Analysis Reveals Disruption of Cholesterol Homeostasis by Cannabidiol in Human Cell Lines. Mol Cell Proteomics. 2022 Oct;21(10):100262. doi: 10.1016/j.mcpro.2022.100262. Epub 2022 Jun 24. PMID: 35753663; PMCID: PMC9525918. |
| Novel Approaches: Targeting Sympathetic Outflow in the Carotid Sinus. | Hering D, Narkiewicz K. Novel approaches: targeting sympathetic outflow in the carotid sinus. Blood Press. 2023 Dec;32(1):2232873. doi: 10.1080/08037051.2023.2232873. PMID: 37470450. |
| Overview of Synthetic Cannabinoids ADB-FUBINACA and AMB-FUBINACA: Clinical, Analytical, and Forensic Implications. [Review] | Lobato-Freitas C, Brito-da-Costa AM, Dinis-Oliveira RJ, Carmo H, Carvalho F, Silva JP, Dias-da-Silva D. Overview of Synthetic Cannabinoids ADB-FUBINACA and AMB-FUBINACA: Clinical, Analytical, and Forensic Implications. Pharmaceuticals (Basel). 2021 Feb 25;14(3):186. doi: 10.3390/ph14030186. PMID: 33669071; PMCID: PMC7996508. |
| Pharmacological Properties of the Plant-Derived Natural products Cannabinoids and Implications for Cardiovascular Health. | Liberale L, Montecucco F, Carbone F. Pharmacological Properties of the Plant-Derived Natural products Cannabinoids and Implications for Cardiovascular Health. Adv Exp Med Biol. 2021;1308:249-255. doi: 10.1007/978-3-030-64872-5_17. PMID: 33861448. |
| Plant-Derived Natural Therapeutics Targeting Cannabinoid Receptors in Metabolic Syndrome and Its Complications: A Review. | Patil AS, Mahajan UB, Agrawal YO, Patil KR, Patil CR, Ojha S, Sharma C, Goyal SN. Plant-derived natural therapeutics targeting cannabinoid receptors in metabolic syndrome and its complications: A review. Biomed Pharmacother. 2020 Dec;132:110889. doi: 10.1016/j.biopha.2020.110889. Epub 2020 Oct 28. PMID: 33113429. |
| Prevention of Cardiovascular Complications of the Metabolic Syndrome: Focus on Pharmacotherapy. | Krentz AJ. Prevention of cardiovascular complications of the metabolic syndrome: focus on pharmacotherapy. Metab Syndr Relat Disord. 2006 Winter;4(4):328-41. doi: 10.1089/met.2006.4.328. PMID: 18370750. |
| Prospects for the Use of Cannabinoid Receptor Ligands for the Treatment of Metabolic Syndrome and Atherosclerosis: Analysis of Experimental and Clinical Data. | Maslov LN, Karpov RS. [Prospects for the Use of Cannabinoid Receptor Ligands for the Treatment of Metabolic Syndrome and Atherosclerosis: Analysis of Experimental and Clinical Data]. Vestn Ross Akad Med Nauk. 2017;72(1):59-65. Russian. doi: 10.15690/vramn779. PMID: 29308855. |
| Rimonabant, a selective cannabinoid CB1 receptor antagonist, inhibits atherosclerosis in LDL receptor-deficient mice. | Dol-Gleizes F, Paumelle R, Visentin V, Marés AM, Desitter P, Hennuyer N, Gilde A, Staels B, Schaeffer P, Bono F. Rimonabant, a selective cannabinoid CB1 receptor antagonist, inhibits atherosclerosis in LDL receptor-deficient mice. Arterioscler Thromb Vasc Biol. 2009 Jan;29(1):12-8. doi: 10.1161/ATVBAHA.108.168757. Epub 2008 Oct 9. PMID: 18845788. |
| Role of Cannabinoid Signaling in Cardiovascular Function and Ischemic Injury | Rorabaugh BR, Guindon J, Morgan DJ. Role of Cannabinoid Signaling in Cardiovascular Function and Ischemic Injury. J Pharmacol Exp Ther. 2023 Dec;387(3):265-276. doi: 10.1124/jpet.123.001665. Epub 2023 Sep 22. PMID: 37739804; PMCID: PMC10658922. |
| Role of the Endocannabinoid System in Metabolic Control Processes and in the Pathogenesis of Metabolic Syndrome: An Update | Dörnyei G, Vass Z, Juhász CB, Nádasy GL, Hunyady L, Szekeres M. Role of the Endocannabinoid System in Metabolic Control Processes and in the Pathogenesis of Metabolic Syndrome: An Update. Biomedicines. 2023 Jan 21;11(2):306. doi: 10.3390/biomedicines11020306. PMID: 36830844; PMCID: PMC9952954. |
| Stilbenoids and cannabinoids from the leaves of Cannabis sativa f. sativa with potential reverse cholesterol transport activity | Guo T , Liu Q , Hou P , Li F , Guo S , Song W , Zhang H , Liu X , Zhang S , Zhang J , Ho CT , Bai N . Stilbenoids and cannabinoids from the leaves of Cannabis sativa f. sativa with potential reverse cholesterol transport activity. Food Funct. 2018 Dec 13;9(12):6608-6617. doi: 10.1039/c8fo01896k. PMID: 30500001. |
| Synthetic Cannabinoids-Related Cardiovascular Emergencies: A Review of the Literature. [Review] | Hasan MR, Tabassum T, Tabassum T, Tanbir MA, Abdelsalam M, Nambiar R. Synthetic Cannabinoids-Related Cardiovascular Emergencies: A Review of the Literature. Cureus. 2023 Jul 15;15(7):e41929. doi: 10.7759/cureus.41929. PMID: 37583720; PMCID: PMC10424760. |
| The effects of acute and sustained cannabidiol dosing for seven days on the haemodynamics in healthy men: A randomised controlled trial | Sultan SR, O'Sullivan SE, England TJ. The effects of acute and sustained cannabidiol dosing for seven days on the haemodynamics in healthy men: A randomised controlled trial. Br J Clin Pharmacol. 2020 Jun;86(6):1125-1138. doi: 10.1111/bcp.14225. Epub 2020 Mar 3. PMID: 32128848; PMCID: PMC7256118. |
| The emerging role of the endocannabinoid system in cardiovascular disease | Pacher P, Steffens S. The emerging role of the endocannabinoid system in cardiovascular disease. Semin Immunopathol. 2009 Jun;31(1):63-77. doi: 10.1007/s00281-009-0145-8. Epub 2009 Apr 9. PMID: 19357846; PMCID: PMC2791499. |
| The Endocannabinoid System in Cardiovascular Function: Novel Insights and Clinical Implications. | Sierra S, Luquin N, Navarro-Otano J. The endocannabinoid system in cardiovascular function: novel insights and clinical implications. Clin Auton Res. 2018 Feb;28(1):35-52. doi: 10.1007/s10286-017-0488-5. Epub 2017 Dec 8. PMID: 29222605. |
| The Influence of Oral Cannabidiol on 24-h Ambulatory Blood Pressure and Arterial Stiffness in Untreated Hypertension: A Double-Blind, Placebo-Controlled, Cross-Over Pilot Study. | Dragun T, Brown CV, Tulppo MP, Obad A, Dujić Ž. The Influence of Oral Cannabidiol on 24-h Ambulatory Blood Pressure and Arterial Stiffness in Untreated Hypertension: A Double-Blind, Placebo-Controlled, Cross-Over Pilot Study. Adv Ther. 2023 Aug;40(8):3495-3511. doi: 10.1007/s12325-023-02560-8. Epub 2023 Jun 8. PMID: 37291376. |
| The Non-Psychoactive Plant Cannabinoid, Cannabidiol Affects Cholesterol Metabolism-Related Genes in Microglial Cells | Rimmerman N, Juknat A, Kozela E, Levy R, Bradshaw HB, Vogel Z. The non-psychoactive plant cannabinoid, cannabidiol affects cholesterol metabolism-related genes in microglial cells. Cell Mol Neurobiol. 2011 Aug;31(6):921-30. doi: 10.1007/s10571-011-9692-3. Epub 2011 Apr 30. PMID: 21533611; PMCID: PMC11498456. |
| The potential use of cannabidiol in the therapy of metabolic syndrome. | Kleiner D, Ditrói K. A kannabidiol gyógyászati alkalmazhatósága metabolikus szindrómában [The potential use of cannabidiol in the therapy of metabolic syndrome]. Orv Hetil. 2012 Apr 1;153(13):499-504. Hungarian. doi: 10.1556/OH.2012.29308. PMID: 22430005. |
| The Role of Acute and Long-Term Use of Cannabinoids on Hypertension and Kidney Injury. | Golosova D, Levchenko V, Kravtsova O, Palygin O, Staruschenko A. Acute and long-term effects of cannabinoids on hypertension and kidney injury. Sci Rep. 2022 Apr 12;12(1):6080. doi: 10.1038/s41598-022-09902-6. PMID: 35413977; PMCID: PMC9005691. |
| The Role of Endocannabinoid System Blockade in the Treatment of the Metabolic Syndrome - Kakafika, AI; Mikhailidis, DP; Athyros, VG (May 2007). | Kakafika AI, Mikhailidis DP, Karagiannis A, Athyros VG. The role of endocannabinoid system blockade in the treatment of the metabolic syndrome. J Clin Pharmacol. 2007 May;47(5):642-52. doi: 10.1177/0091270007299358. Epub 2007 Mar 28. PMID: 17392496. |
| The role of the endocannabinoid system in atherosclerosis. | Mach F, Steffens S. The role of the endocannabinoid system in atherosclerosis. J Neuroendocrinol. 2008 May;20 Suppl 1:53-7. doi: 10.1111/j.1365-2826.2008.01685.x. PMID: 18426500. |
| The Use of Cannabidiol in Metabolic Syndrome-An Opportunity to Improve the Patient's Health or Much Ado about Nothing? | Wiciński M, Fajkiel-Madajczyk A, Kurant Z, Gryczka K, Kurant D, Szambelan M, Malinowski B, Falkowski M, Zabrzyński J, Słupski M. The Use of Cannabidiol in Metabolic Syndrome-An Opportunity to Improve the Patient's Health or Much Ado about Nothing? J Clin Med. 2023 Jul 11;12(14):4620. doi: 10.3390/jcm12144620. PMID: 37510734; PMCID: PMC10380672. |
| Therapeutic potential of cannabinoid-based drugs | Klein TW, Newton CA. Therapeutic potential of cannabinoid-based drugs. Adv Exp Med Biol. 2007;601:395-413. doi: 10.1007/978-0-387-72005-0_43. PMID: 17713029. |
| Towards a therapeutic use of selective CB2 cannabinoid receptor ligands for atherosclerosis. | Steffens S, Mach F. Towards a therapeutic use of selective CB2 cannabinoid receptor ligands for atherosclerosis. Future Cardiol. 2006 Jan;2(1):49-53. doi: 10.2217/14796678.2.1.49. PMID: 19804131. |
| Treatment with Cannabidiol Results in an Antioxidant and Cardioprotective Effect in Several Pathophysiologies. | Oliveira NMC, Machado DA, da Silva TL, do Vale GT. Treatment with Cannabidiol Results in an Antioxidant and Cardioprotective Effect in Several Pathophysiologies. Curr Hypertens Rev. 2022;18(2):125-129. doi: 10.2174/1573402118666220513164101. PMID: 36508270. |
| Trial of a Novel Oral Cannabinoid Formulation in Patients with Hypertension: A Double-Blind, Placebo-Controlled Pharmacogenetic Study* | Batinic A, Sutlović D, Kuret S, Matana A, Kumric M, Bozic J, Dujic Z. Trial of a Novel Oral Cannabinoid Formulation in Patients with Hypertension: A Double-Blind, Placebo-Controlled Pharmacogenetic Study. Pharmaceuticals (Basel). 2023 Apr 25;16(5):645. doi: 10.3390/ph16050645. PMID: 37242428; PMCID: PMC10223705. |
| Why Multitarget Vasodilatory (Endo)cannabinoids Are Not Effective as Antihypertensive Compounds after Chronic Administration: Comparison of Their Effects on Systemic and Pulmonary Hypertension. [Review] | Remiszewski P, Malinowska B. Why Multitarget Vasodilatory (Endo)cannabinoids Are Not Effective as Antihypertensive Compounds after Chronic Administration: Comparison of Their Effects on Systemic and Pulmonary Hypertension. Pharmaceuticals (Basel). 2022 Sep 7;15(9):1119. doi: 10.3390/ph15091119. PMID: 36145339; PMCID: PMC9503677. |
