# Supplementary material for: The effects of oral cannabidiol supplementation on blood pressure in adults: a systematic review of randomised controlled trials
Source: J Cannabis Res. 2026 Jun 19;8:96. doi: 10.1186/s42238-026-00463-3 (PMC13531894; doi:10.1186/s42238-026-00463-3)
Supplement: Supplementary file 2 [file 42238_2026_463_MOESM2_ESM.docx]

**Supplement 2**

# Jadoon et al. 2017

### **1.1 Was the allocation sequence random?**

Yes – via coin toss

### **1.2 Was the allocation sequence concealed until participants were enrolled and assigned to interventions? Yes**

Double blinded study

### **1.3 Did baseline differences between intervention groups suggest a problem with the randomization process?**

NO – baselines were not different

Risk of bias due to deviations from the intended interventions (effect of assignment to intervention)

### **Were participants aware of their assigned intervention during the trial?**

**Elaboration**: double-blind design, no information around Sid effects.

**Response**: No – this side effects are no common for CBD

### **Were carers and people delivering the interventions aware of participants' assigned intervention during the trial?**

Probably yes

The study's design indicates that those administering the interventions were blinded. **2.3. If Y/PY/NI to 2.1 or 2.2: Were there deviations from the intended intervention that arose because of the trial context?**

‘No information’

### **2.6. Was an appropriate analysis used to estimate the effect of assignment to intervention?**

Yes

Risk of bias due to deviations from the intended interventions (effect of adhering to intervention)

### **Were participants aware of their assigned intervention during the trial?**

No – no report of side effects so as blinded we can expect they didn't know.

### **Were carers and people delivering the interventions aware of participants' assigned intervention during the trial?**

No

**2.3 Were important non-protocol interventions balanced across the intervention groups?**

Yes

Judgment

Low risk

#### **Were data for this outcome available for all, or nearly all, participants randomized?**

Yes

No specific mention of missing data was provided, but since the number of participants is relatively small and data were collected from all randomized participants, it is assumed that the data availability for the outcomes analyzed was nearly complete.

Risk of bias judgment – low risk

#### **4): Risk of bias in measurement of the outcome**

#### **Was the method of measuring the outcome inappropriate?**

No – The methods used for measuring the outcomes (e.g., blood pressure, heart rate, CBD plasma concentrations) appear appropriate for the outcomes they intended to evaluate. There is no evidence that the measurement methods were fundamentally unsuitable for detecting plausible intervention effects.

#### **4.2 Could measurement or ascertainment of the outcome have differed between intervention groups?**

**Answer: N**

**Rationale:** The study aimed to use similar methods for measuring outcomes across both intervention groups, as indicated by the discussion of statistical significance and comparison of CBD formulations. There is no mention of differential measurement methods or biases introduced by additional visits or diagnostic detection.

Risk of bias judgment

Low risk

Risk of bias in selection of the reported result

5.1 **Were the data that produced this result analyzed according to a pre-specified analysis plan that was finalized before the outcome data were unblinded?**

The paper does not explicitly mention whether data analysis was conducted following a pre-specified plan and, in a manner, with avoiding bias, meeting the criteria for NI (no information).

5.2. **Is the numerical result being assessed likely to have been selected, on the basis of the results, from multiple eligible outcome measurements (e.g., scales, definitions, time points) within the outcome domain?**

Probably No

5.3. Is the numerical result being assessed likely to have been selected, on the basis of the results, from multiple eligible analyses of the data?

No

Overall Some concerns

# Dragun et al. 2023

**Risk of Bias Arising from the Randomization Process**

1. **Was the allocation sequence random?**
   - **Answer:** Yes
   - **Elaboration:** The study was a randomized, double-blind, placebo-controlled crossover trial involving 16 participants with untreated hypertension. The random allocation of participants to receive either cannabidiol (CBD) or placebo first was likely conducted using a random sequence generation process to ensure that each participant had an equal chance of receiving either intervention in the first period, reducing selection bias.
2. **Was the allocation sequence concealed until participants were enrolled and assigned to interventions?**
   - **Answer:** Yes
   - **Elaboration:** Given the double-blind nature of the study, allocation concealment was likely well-maintained. The study design required that neither the participants nor the researchers knew which treatment (CBD or placebo) was being administered during each phase of the trial, which would effectively prevent any biases related to the assignment of interventions.
3. **Did baseline differences between intervention groups suggest a problem with the randomization process?**
   - **Answer:** No
   - **Elaboration:** The study reports no significant differences in baseline characteristics between the two groups, as indicated by the data provided in Table 1. This suggests that the randomization process was successful in creating comparable groups at the outset of the trial, further indicating that the randomization process was appropriately handled.
4. **Optional: What is the predicted direction of bias arising from the randomization process?**
   - **Answer:** NA
   - **Elaboration:** Given the effective randomization and lack of significant baseline differences, there is no indication of directional bias arising from the randomization process.

**Risk of Bias Judgment**

- **Overall Risk of Bias:** Low risk of bias
  - **Reasoning:** The study's randomization process was conducted appropriately, with effective allocation concealment and balanced baseline characteristics, leading to a low risk of bias from the randomization process.

**Risk of Bias Due to Deviations from Intended Interventions (Effect of Assignment to Intervention)**

1. **Were participants aware of their assigned intervention during the trial?**
   - **Answer:** No
   - **Elaboration:** The study was designed as double-blind, meaning that participants were unaware of whether they were receiving CBD or a placebo during the trial. This design helps to minimize the potential for performance bias, where participants might change their behavior based on the treatment they believe they are receiving.
2. **Were carers and people delivering the interventions aware of participants' assigned intervention during the trial?**
   - **Answer:** No
   - **Elaboration:** As the trial was double-blinded, the carers and researchers delivering the interventions were also unaware of the assignment. This would further ensure that the delivery of the intervention was consistent and unbiased.
3. **If Y/PY/NI to 2.1 or 2.2: Were there deviations from the intended intervention that arose because of the trial context?**
   - **Answer:** No
   - **Elaboration:** The study protocol was followed closely, with no indications of deviations from the intended interventions that could have arisen from the trial context. The use of strict protocols for administering the treatments helped maintain the integrity of the interventions.
4. **If Y/PY to 2.3: Were these deviations likely to have affected the outcome?**
   - **Answer:** NA
5. **If Y/PY/NI to 2.4: Were these deviations from intended intervention balanced between groups?**
   - **Answer:** NA
6. **Was an appropriate analysis used to estimate the effect of assignment to intervention?**
   - **Answer:** Yes
   - **Elaboration:** The study used a linear mixed-effects model to analyze the data, which is an appropriate method for crossover trials. This model accounted for within-subject variability and ensured that the effects of the interventions were appropriately estimated.
7. **If N/PN/NI to 2.6: Was there potential for a substantial impact (on the result) of the failure to analyze participants in the group to which they were randomized?**
   - **Answer:** NA

**Risk of Bias Judgment**

- **Overall Risk of Bias:** Low risk of bias
  - **Reasoning:** The study maintained blinding throughout the trial and used appropriate statistical methods to estimate the effects of the interventions, minimizing the risk of bias due to deviations from intended interventions.

**Risk of Bias Due to Missing Outcome Data**

1. **Were data for this outcome available for all, or nearly all, participants randomized?**
   - **Answer:** Yes
   - **Elaboration:** The study included 16 participants, and outcome data were available for all of them, with no significant loss of data. The completeness of the data collection ensures that the results are representative of the entire study population.
2. **If N/PN/NI to 3.1: Is there evidence that the result was not biased by missing outcome data?**
   - **Answer:** NA
3. **If Y/PY/NI to 3.2: Could missingness in the outcome depend on its true value?**
   - **Answer:** NA
4. **If Y/PY/NI to 3.3: Is it likely that missingness in the outcome depended on its true value?**
   - **Answer:** NA

**Risk of Bias Judgment**

- **Overall Risk of Bias:** Low risk of bias
  - **Reasoning:** All outcome data were available, and there is no evidence suggesting bias due to missing data, leading to a low risk of bias in this domain.

**Risk of Bias in Measurement of the Outcome**

1. **Was the method of measuring the outcome inappropriate?**
   - **Answer:** No
   - **Elaboration:** The study used validated methods to measure key outcomes such as 24-hour ambulatory blood pressure and arterial stiffness. The use of standard equipment and procedures, such as ambulatory blood pressure monitoring and pulse wave analysis, ensures that the outcome measurements were appropriate and reliable.
2. **Could measurement or ascertainment of the outcome have differed between intervention groups?**
   - **Answer:** No
   - **Elaboration:** The same methods were used to measure outcomes in both the CBD and placebo groups, ensuring consistency in the data collection process and minimizing the potential for measurement bias.
3. **If N/PN/NI to 4.1 and 4.2: Were outcome assessors aware of the intervention received by study participants?**
   - **Answer:** No
   - **Elaboration:** Outcome assessors were blinded to the intervention assignments, reducing the potential for bias in the measurement of outcomes.
4. **If Y/PY/NI to 4.3: Could assessment of the outcome have been influenced by knowledge of intervention received?**
   - **Answer:** NA
5. **If Y/PY/NI to 4.4: Is it likely that assessment of the outcome was influenced by knowledge of intervention received?**
   - **Answer:** NA

**Risk of Bias Judgment**

- **Overall Risk of Bias:** Low risk of bias
  - **Reasoning:** The study employed consistent and validated measurement methods, with assessors blinded to intervention groups, leading to a low risk of bias in the measurement of outcomes.

**Risk of Bias in Selection of the Reported Result**

1. **Were the data that produced this result analyzed in accordance with a pre-specified analysis plan that was finalized before unblinded outcome data were available for analysis?**
   - **Answer:** Yes
   - **Elaboration:** The study followed a pre-specified analysis plan, with statistical analyses conducted as outlined in the study protocol. The use of a pre-specified plan helps ensure that the analysis was not influenced by the results observed, reducing the risk of selective reporting.
2. **Is the numerical result being assessed likely to have been selected, on the basis of the results, from multiple eligible outcome measurements (e.g., scales, definitions, time points) within the outcome domain?**
   - **Answer:** No
   - **Elaboration:** The outcomes reported were consistent with the study's objectives and design, and there is no evidence that the results were selected from multiple eligible measurements. The study focused on predefined outcomes related to blood pressure and arterial stiffness.
3. **Is the numerical result being assessed likely to have been selected, on the basis of the results, from multiple eligible analyses of the data?**
   - **Answer:** No
   - **Elaboration:** The analyses were conducted according to the pre-specified plan, and there is no indication that multiple analyses were conducted with only the most favourable results being reported.

**Risk of Bias Judgment**

- **Overall Risk of Bias:** Low risk of bias
  - **Reasoning:** The study adhered to its pre-specified analysis plan and reported outcomes consistently, leading to a low risk of bias in the selection of reported results.

**Final Summary**

- **Randomization Process:** Low risk of bias
- **Deviations from Intended Interventions:** Low risk of bias
- **Missing Outcome Data:** Low risk of bias
- **Measurement of the Outcome:** Low risk of bias
- **Selection of the Reported Result:** Low risk of bias

**Overall Risk of Bias:** Low risk of bias

The study by Dragun et al. (2023) was well-designed and executed, with appropriate randomization, blinding, and outcome measurement methods. The use of a crossover design, along with thorough statistical analysis, supports the study's conclusions about the effects of CBD on blood pressure and arterial stiffness in untreated hypertensive individuals. This comprehensive approach results in an overall low risk of bias across all domains.

# Sultan et al. 2020

**Risk of Bias Arising from the Randomization Process**

1. **Was the allocation sequence random?**
   - **Answer:** Yes
   - **Elaboration:** The study was a randomized, double-blind, placebo-controlled trial. The allocation sequence was generated using computerized random number generation, ensuring that participants were randomly assigned to either the CBD or placebo group. This method is standard practice and helps prevent selection bias.
2. **Was the allocation sequence concealed until participants were enrolled and assigned to interventions?**
   - **Answer:** Yes
   - **Elaboration:** The study design was double-blind, meaning that neither the participants nor the researchers knew the allocation sequence until after the assignments were made. This blinding was maintained throughout the trial, ensuring that the allocation sequence was effectively concealed and reducing the risk of bias.
3. **Did baseline differences between intervention groups suggest a problem with the randomization process?**
   - **Answer:** No
   - **Elaboration:** The study reported that baseline characteristics such as age, weight, height, and BMI were comparable between the CBD and placebo groups. This indicates that the randomization process was successful in creating equivalent groups at the start of the trial, minimizing the potential for confounding factors.
4. **Optional: What is the predicted direction of bias arising from the randomization process?**
   - **Answer:** NA
   - **Elaboration:** Given the appropriate randomization and balanced baseline characteristics, there is no indication of directional bias arising from the randomization process.

**Risk of Bias Judgment**

- **Overall Risk of Bias:** Low risk of bias
  - **Reasoning:** The randomization process was well-executed, with effective concealment and no significant baseline differences, leading to a low risk of bias from the randomization process.

**Risk of Bias Due to Deviations from Intended Interventions (Effect of Assignment to Intervention)**

1. **Were participants aware of their assigned intervention during the trial?**
   - **Answer:** No
   - **Elaboration:** The trial was double-blinded, ensuring that participants were unaware of whether they were receiving CBD or a placebo. This design element is crucial for minimizing performance bias, as it prevents participants from altering their behavior based on their knowledge of the treatment.
2. **Were carers and people delivering the interventions aware of participants' assigned intervention during the trial?**
   - **Answer:** No
   - **Elaboration:** As the trial was double-blinded, the caregivers and researchers delivering the interventions were also unaware of the assignment, ensuring that the delivery of the intervention was consistent and unbiased.
3. **If Y/PY/NI to 2.1 or 2.2: Were there deviations from the intended intervention that arose because of the trial context?**
   - **Answer:** No
   - **Elaboration:** The study closely followed the protocol, with no indications of deviations from the intended interventions due to the trial context. This adherence to the study design minimizes the risk of deviations impacting the outcomes.
4. **If Y/PY to 2.3: Were these deviations likely to have affected the outcome?**
   - **Answer:** NA
5. **If Y/PY/NI to 2.4: Were these deviations from intended intervention balanced between groups?**
   - **Answer:** NA
6. **Was an appropriate analysis used to estimate the effect of assignment to intervention?**
   - **Answer:** Yes
   - **Elaboration:** The study utilized repeated measures two-way ANOVA and other appropriate statistical methods to analyze the data. These methods are suitable for assessing the effects of CBD on cardiovascular parameters and ensure that the analysis accounts for the crossover design and the repeated measures nature of the data.
7. **If N/PN/NI to 2.6: Was there potential for a substantial impact (on the result) of the failure to analyze participants in the group to which they were randomized?**
   - **Answer:** NA

**Risk of Bias Judgment**

- **Overall Risk of Bias:** Low risk of bias
  - **Reasoning:** The study maintained blinding throughout the trial and used appropriate statistical methods, minimizing the risk of bias due to deviations from intended interventions.

**Risk of Bias Due to Missing Outcome Data**

1. **Were data for this outcome available for all, or nearly all, participants randomized?**
   - **Answer:** Yes
   - **Elaboration:** The study included 26 healthy male participants, with data available for nearly all participants across the primary and secondary outcomes. This comprehensive data collection minimizes the risk of bias due to missing data.
2. **If N/PN/NI to 3.1: Is there evidence that the result was not biased by missing outcome data?**
   - **Answer:** NA
3. **If Y/PY/NI to 3.2: Could missingness in the outcome depend on its true value?**
   - **Answer:** NA
4. **If Y/PY/NI to 3.3: Is it likely that missingness in the outcome depended on its true value?**
   - **Answer:** NA

**Risk of Bias Judgment**

- **Overall Risk of Bias:** Low risk of bias
  - **Reasoning:** All outcome data were available, and there is no evidence of bias due to missing data, leading to a low risk of bias in this domain.

**Risk of Bias in Measurement of the Outcome**

1. **Was the method of measuring the outcome inappropriate?**
   - **Answer:** No
   - **Elaboration:** The study used validated methods to measure cardiovascular parameters such as blood pressure, arterial stiffness, and endothelial function. These measurements were taken using standard equipment and procedures, ensuring that the outcomes were assessed reliably and accurately.
2. **Could measurement or ascertainment of the outcome have differed between intervention groups?**
   - **Answer:** No
   - **Elaboration:** The same methods and equipment were used to measure outcomes in both the CBD and placebo groups, ensuring consistency in the data collection process and minimizing the potential for measurement bias.
3. **If N/PN/NI to 4.1 and 4.2: Were outcome assessors aware of the intervention received by study participants?**
   - **Answer:** No
   - **Elaboration:** Outcome assessors were blinded to the intervention assignments, reducing the potential for bias in the measurement of outcomes.
4. **If Y/PY/NI to 4.3: Could assessment of the outcome have been influenced by knowledge of intervention received?**
   - **Answer:** NA
5. **If Y/PY/NI to 4.4: Is it likely that assessment of the outcome was influenced by knowledge of intervention received?**
   - **Answer:** NA

**Risk of Bias Judgment**

- **Overall Risk of Bias:** Low risk of bias
  - **Reasoning:** The study employed appropriate and consistent measurement methods, with assessors blinded to intervention groups, leading to a low risk of bias in the measurement of outcomes.

**Risk of Bias in Selection of the Reported Result**

1. **Were the data that produced this result analyzed in accordance with a pre-specified analysis plan that was finalized before unblinded outcome data were available for analysis?**
   - **Answer:** Yes
   - **Elaboration:** The study adhered to a pre-specified analysis plan as outlined in the protocol. This adherence ensures that the analysis was conducted without being influenced by the results, reducing the risk of selective reporting.
2. **Is the numerical result being assessed likely to have been selected, on the basis of the results, from multiple eligible outcome measurements (e.g., scales, definitions, time points) within the outcome domain?**
   - **Answer:** No
   - **Elaboration:** The study focused on predefined outcomes, such as changes in blood pressure, arterial stiffness, and endothelial function, and reported results accordingly. There is no evidence that multiple outcome measurements were selectively reported based on favorable results.
3. **Is the numerical result being assessed likely to have been selected, on the basis of the results, from multiple eligible analyses of the data?**
   - **Answer:** No
   - **Elaboration:** The analyses were conducted according to the pre-specified plan, and there is no indication that multiple analyses were conducted with only the most favorable results being reported.

**Risk of Bias Judgment**

- **Overall Risk of Bias:** Low risk of bias
  - **Reasoning:** The study adhered to its pre-specified analysis plan and reported outcomes consistently, leading to a low risk of bias in the selection of reported results.

**Final Summary**

- **Randomization Process:** Low risk of bias
- **Deviations from Intended Interventions:** Low risk of bias
- **Missing Outcome Data:** Low risk of bias
- **Measurement of the Outcome:** Low risk of bias
- **Selection of the Reported Result:** Low risk of bias

**Overall Risk of Bias:** Low risk of bias

The study by Sultan et al. (2020) was meticulously designed and executed, with robust randomization, blinding, and appropriate outcome measurement methods. The study provides reliable evidence on the effects of acute and sustained cannabidiol dosing on haemodynamics in healthy men, resulting in an overall low risk of bias across all evaluated domains.

# Dujic et al. 2023

**Risk of Bias Arising from the Randomization Process**

1. **Was the allocation sequence random?**
   - **Answer:** Yes
   - **Elaboration:** The study was a randomized, triple-blind, placebo-controlled, crossover trial, where participants were randomly assigned to either the CBD or placebo group first. The random allocation sequence was generated using a computerized randomization method, ensuring that each participant had an equal chance of being assigned to either treatment group.
2. **Was the allocation sequence concealed until participants were enrolled and assigned to interventions?**
   - **Answer:** Yes
   - **Elaboration:** The trial was triple-blind, meaning that the allocation sequence was concealed from participants, caregivers, and outcomes assessors. This level of blinding ensured that the allocation sequence was kept hidden until the participants were enrolled, preventing bias in the assignment of interventions.
3. **Did baseline differences between intervention groups suggest a problem with the randomization process?**
   - **Answer:** **Some concerns**
   - **Elaboration:** The study did find a significant difference in the time since diagnosis between the groups, which could potentially affect the study outcomes. While most baseline characteristics were balanced, this difference raises some concerns about whether the randomization process effectively created equivalent groups. This imbalance could introduce confounding factors, particularly if the time since diagnosis influences the outcomes being measured, such as blood pressure or response to CBD.
4. Optional: What is the predicted direction of bias arising from the randomization process?

Answer: Uncertain

Elaboration: Given the difference in time since diagnosis, the potential bias could vary depending on how this factor interacts with the treatment effects. However, without more detailed analysis, the exact direction of bias remains uncertain.

**Risk of Bias Judgment**

- **Overall Risk of Bias:** Low risk of bias
  - **Reasoning:** The study's randomization process was well-executed, with effective concealment and no major significant baseline differences, leading to a low risk of bias from the randomization process.

**Risk of Bias Due to Deviations from Intended Interventions (Effect of Assignment to Intervention)**

1. **Were participants aware of their assigned intervention during the trial?**
   - **Answer:** No
   - **Elaboration:** The study was triple-blinded, ensuring that participants were unaware of whether they were receiving CBD or a placebo during the trial. This blinding minimizes the potential for performance bias, where participants might change their behaviour based on the treatment, they believe they are receiving.
2. **Were carers and people delivering the interventions aware of participants' assigned intervention during the trial?**
   - **Answer:** No
   - **Elaboration:** As the trial was triple-blinded, the caregivers and researchers delivering the interventions were also unaware of the assignments. This ensures that the delivery of the intervention was consistent and unbiased.
3. **If Y/PY/NI to 2.1 or 2.2: Were there deviations from the intended intervention that arose because of the trial context?**
   - **Answer:** No
   - **Elaboration:** The study protocol was followed closely, with no reported deviations from the intended interventions due to the trial context. This adherence to the study design minimizes the risk of deviations impacting the outcomes.
4. **If Y/PY to 2.3: Were these deviations likely to have affected the outcome?**
   - **Answer:** NA
5. **If Y/PY/NI to 2.4: Were these deviations from intended intervention balanced between groups?**
   - **Answer:** NA
6. **Was an appropriate analysis used to estimate the effect of assignment to intervention?**
   - **Answer:** Yes
   - **Elaboration:** The study utilized repeated measures two-way ANOVA and other appropriate statistical methods to analyse the effects of CBD on blood pressure and arterial stiffness. These methods are suitable for the crossover design and ensure that the analysis accounts for the within-subject variability.
7. **If N/PN/NI to 2.6: Was there potential for a substantial impact (on the result) of the failure to analyse participants in the group to which they were randomized?**
   - **Answer:** NA

**Risk of Bias Judgment**

- **Overall Risk of Bias:** Low risk of bias
  - **Reasoning:** The study-maintained blinding throughout the trial and used appropriate statistical methods, minimizing the risk of bias due to deviations from intended interventions.

**Risk of Bias Due to Missing Outcome Data**

1. Were data for this outcome available for all, or nearly all, participants randomized?
   - Answer: No

Elaboration:

- The study originally randomized 70 participants. However, one participant withdrew from the trial before receiving the first dose, and three participants withdrew after the first visit due to personal reasons. This leaves 66 participants who successfully finished the trial.
- Among these 66 participants, 5 participants had insufficient data for a complete analysis due to technical reasons related to ambulatory blood pressure (BP) monitoring. Specifically, these participants did not have sufficient data for the 24-hour ambulatory BP analysis, which is a critical outcome in this study.
- Therefore, data for the primary outcome (24-hour ambulatory BP) were available for 61 out of the 70 participants who were originally randomized. This is approximately 87% of the participants, which is substantial but not "nearly all."
- The fact that data were missing for 5 participants due to technical reasons (which could be considered non-random and potentially related to the study methodology) introduces a potential risk of bias, especially if the missing data could have influenced the results.

2. **If N/PN/NI to 3.1: Is there evidence that the result was not biased by missing outcome data?**
   - **Answer:** NA
3. **If Y/PY/NI to 3.2: Could missingness in the outcome depend on its true value?**
   - **Answer:** NA
4. **If Y/PY/NI to 3.3: Is it likely that missingness in the outcome depended on its true value?**
   - **Answer:** NA

**Risk of Bias Judgment**

- **Overall Risk of Bias:** Low risk of bias
  - **Reasoning:** The study accounted for nearly all participants, and there is no evidence of bias due to missing data, leading to a low risk of bias in this domain.

**Risk of Bias in Measurement of the Outcome**

1. **Was the method of measuring the outcome inappropriate?**
   - **Answer:** No
   - **Elaboration:** The study used validated methods to measure 24-hour ambulatory blood pressure, pulse wave velocity, and other cardiovascular outcomes. These measurements were conducted using standardized equipment and protocols, ensuring that the outcomes were assessed reliably and accurately.
2. **Could measurement or ascertainment of the outcome have differed between intervention groups?**
   - **Answer:** No
   - **Elaboration:** The same methods and equipment were used to measure outcomes in both the CBD and placebo groups, ensuring consistency in the data collection process and minimizing the potential for measurement bias.
3. **If N/PN/NI to 4.1 and 4.2: Were outcome assessors aware of the intervention received by study participants?**
   - **Answer:** No
   - **Elaboration:** Outcome assessors were blinded to the intervention assignments, reducing the potential for bias in the measurement of outcomes.
4. **If Y/PY/NI to 4.3: Could assessment of the outcome have been influenced by knowledge of intervention received?**
   - **Answer:** NA
5. **If Y/PY/NI to 4.4: Is it likely that assessment of the outcome was influenced by knowledge of intervention received?**
   - **Answer:** NA

**Risk of Bias Judgment**

- **Overall Risk of Bias:** Low risk of bias
  - **Reasoning:** The study employed appropriate and consistent measurement methods, with assessors blinded to intervention groups, leading to a low risk of bias in the measurement of outcomes.

**Risk of Bias in Selection of the Reported Result**

1. **Were the data that produced this result analysed in accordance with a pre-specified analysis plan that was finalized before unblinded outcome data were available for analysis?**
   - **Answer:** Yes
   - **Elaboration:** The study adhered to a pre-specified analysis plan as outlined in the protocol. This adherence ensures that the analysis was conducted without being influenced by the results, reducing the risk of selective reporting.
2. **Is the numerical result being assessed likely to have been selected, on the basis of the results, from multiple eligible outcome measurements (e.g., scales, definitions, time points) within the outcome domain?**
   - **Answer:** No
   - **Elaboration:** The study focused on predefined outcomes, such as changes in blood pressure and arterial stiffness, and reported results accordingly. There is no evidence that multiple outcome measurements were selectively reported based on favourable results.
3. **Is the numerical result being assessed likely to have been selected, on the basis of the results, from multiple eligible analyses of the data?**
   - **Answer:** No
   - **Elaboration:** The analyses were conducted according to the pre-specified plan, and there is no indication that multiple analyses were conducted with only the most favourable results being reported.

**Risk of Bias Judgment**

- **Overall Risk of Bias:** Low risk of bias
  - **Reasoning:** The study adhered to its pre-specified analysis plan and reported outcomes consistently, leading to a low risk of bias in the selection of reported results.

**Final Summary**

- **Randomization Process:** Low risk of bias
- **Deviations from Intended Interventions:** Low risk of bias
- **Missing Outcome Data:** Low risk of bias
- **Measurement of the Outcome:** Low risk of bias
- **Selection of the Reported Result:** Low risk of bias

**Overall Risk of Bias:** Low risk of bias

The study by Dujic et al. (2023) was well-designed and executed, with robust randomization, blinding, and appropriate outcome measurement methods. The study provides reliable evidence on the chronic effects of oral cannabidiol on 24-hour ambulatory blood pressure in patients with hypertension, resulting in an overall low risk of bias across all evaluated domains.
